# Supplementary material for: Expression of E-cadherin by CD8+ T cells promotes their invasion into biliary epithelial cells
Source: Nat Commun. 2024 Jan 29;15:853. doi: 10.1038/s41467-024-44910-2 (PMC10825166; doi:10.1038/s41467-024-44910-2)
Supplement: Supplementary file 1 — Supplementary information [file 41467_2024_44910_MOESM1_ESM.pdf]

Expression of E-cadherin by CD8$^+$ T cells promotes their invasion into biliary epithelial cells

*Scott P Davies[1, 2, 3, 4, 8], Vincenzo Ronca[1, 2, 4, 8] , Grace E Wootton[1, 2, 3, 4, 7], Natalia M Krajewska[1, 2], Amber G Bozward[1, 2, 3, 4, 7], Rémi Fiancette[1, 2, 3], Daniel A Patten[1, 2, 3], Katharina Yankouskaya[1, 2], Gary M Reynolds[1, 2], Sofia Pat[1, 2], Daniel C Osei-Bordom[1, 2], Naomi Richardson[1, 2, 3, 4, 7], Liam M Grover[2, 5, 6], Christopher J Weston[1, 2. 3], and *$Ye H Oo[1, 2, 3, 4, 7]

1. Centre for Liver and Gastrointestinal Research, Institute of Biomedical Research, Institute of Immunology and Immunotherapy, University of Birmingham, Birmingham, UK
2. National Institute of Health Research Birmingham Biomedical Research Centre, University of Birmingham and University Hospitals Birmingham NHS Foundation Trust, Birmingham, UK
3. National Institute for Health Research, Birmingham Biomedical Research Centre, University Hospitals Birmingham NHS Foundation Trust, Birmingham, UK.
4. European Reference Network on Hepatological Diseases (ERN Rare-Liver), Birmingham, UK
5. School of Chemical Engineering, University of Birmingham, UK
6. Healthcare Technologies Institute, University of Birmingham, Birmingham, UK
7. Birmingham Advanced Cellular Therapy Facility, University of Birmingham, Birmingham, UK
8. Joint first author.

$ Senior author
* Corresponding authors:

Scott P Davies – s.p.davies.1@bham.ac.uk; Ye H Oo – y.h.oo@bham.ac.uk.

SUPPLEMENTARY INFORMATION

SUPPLEMENTARY DISCUSSION

IHC staining of liver tissues demonstrated the persistent expression of CD103 and CD69 by $CD8^+$ T cells internalised within BEC *in vivo.* The co-expression of these residency markers and increased frequency of their internalisation into BEC that we observed *in vitro,* following 48 h TCR stimulation, suggests that the capacity to invade BEC is correlated with the acquisition of hepatic tissue resident phenotype. This is further evidenced by the absence of $KLRG1^+$ $CD8^+$ T cells found within BEC across all liver tissue sections studied. However, due to the proximity of $CD103^+$ $CD69^+$ $CD8^+$ T cells to epithelial surfaces of bile ducts observed *in vivo*, it may be more accurate to call these cells IELs as opposed to a truly resident memory population. Analysis of previously generated murine $CD8^+$ T cell RNAseq data showed clustering of liver-derived $CD8^+$ T cells with small intestine-derived IELs[1]. Further interrogation of this data also revealed that both populations displayed elevated levels of *Cdh1* transcription, and E-cadherin$^+$ $CD8^+$ T cells in mammalian salivary glands were similar in phenotype to gut intraepithelial lymphocytes (IEL) at the protein level[2]. Other studies report the presence of $CD103^+$ $CD8^+$ T cells close to bile ducts[3], and support our finding that E-cadherin expression itself is also linked to the phenotype of IELs. It is not yet clear if E-cadherin$^+$ IELs or other $CD8^+$ T cells can invade the cells comprising their epithelium of residence, or how this may be associated with non-liver autoimmune disease. Additionally, the signals that are required to induce E-cadherin expression on $CD8^+$ T cells *in vivo* and the location at which they acquire E-cadherin are uncertain. Deeper phenotyping into E-cadherin$^+$ $CD103^+$ $CD69^+$ $CD8^+$ T cells found in the liver and other organs will be needed to truly classify this cell subset and better define their functional role.

SUPPLEMENTARY DATA

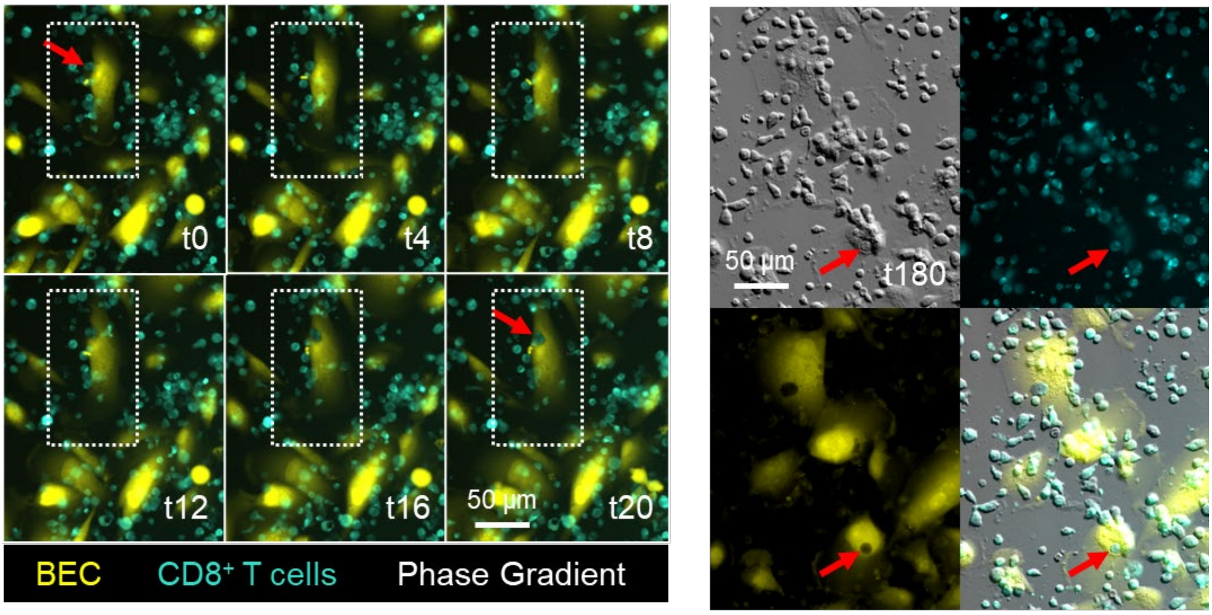

Figure 1. CD8⁺ T cells can be internalised into BEC within 20 min of attachment and remain internalised over a longer time. *Left*: Representative time-lapse images captured at 4 min intervals showing co-cultured biliary epithelial cells (CellTracker™ Green; yellow) and peripheral blood-derived $CD8^+$ T cells (CellTracker™ Red; cyan) 1 h after initial co-culture. T cells were activated with α-CD3/CD28 stimulation and cultured for 48 h prior to their co-culture with BEC. Red arrow shows a $CD8^+$ T cell being internalised throughout the time course. *Right*: Same internalised $CD8^+$ T cell (red arrow) after 180 min of co-culture. Phase Gradient (grey) microscopy images were acquired simultaneously.

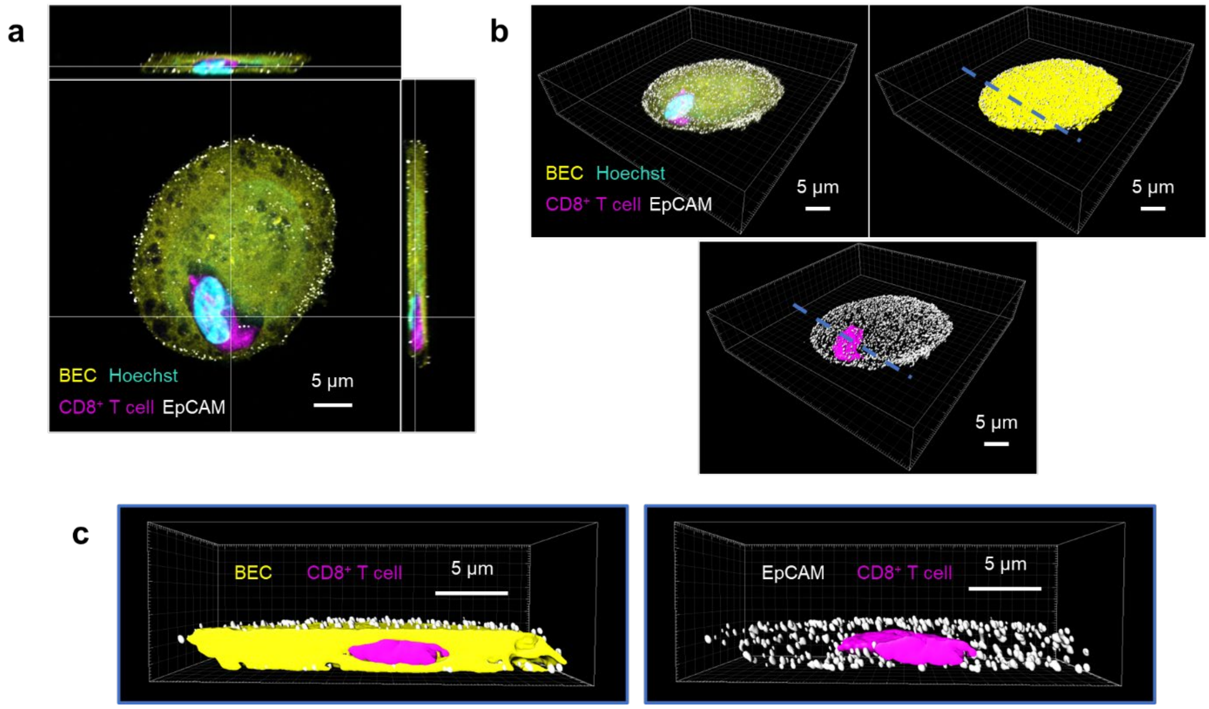

Figure 2. Internalised CD8$^+$ T cells remain within BEC after they have been detached from their culture vessels. Blood-derived CD8$^+$ T cells were activated with α-CD3/CD28 stimulation for 48 h. Cells were then labelled with CellTracker™ Red and co-cultured with CellTracker™ Green-labelled primary human biliary epithelial cells (BEC) for 4 h. Cells were then detached from culture plates with TrypLE™ Express and stained by immunocytochemistry (ICC). Cells were then resuspended in mounting medium and dispensed on to a microscope slide. A cover slip was applied and then cells were imaged using confocal microscopy. **A.** Orthographical Airyscan confocal micrograph showing CD8$^+$ T cell (CellTracker™ Red, magenta) internalised within a BEC (CellTracker™ Green; yellow) stained for EpCAM (grey). Bright nuclear staining (Hoechst; cyan) corresponds to the internalised CD8$^+$ T cell. **B**. 3D-reconstruction of **A** showing 3D-volume rendered versions of BEC cytoplasm (*top right*) and EpCAM staining (*bottom*) in relation to the internalised T cell. **C.** Cross-section views of rendered reconstructions shown in **B**, cut along the blue dotted lines shown in this panel.

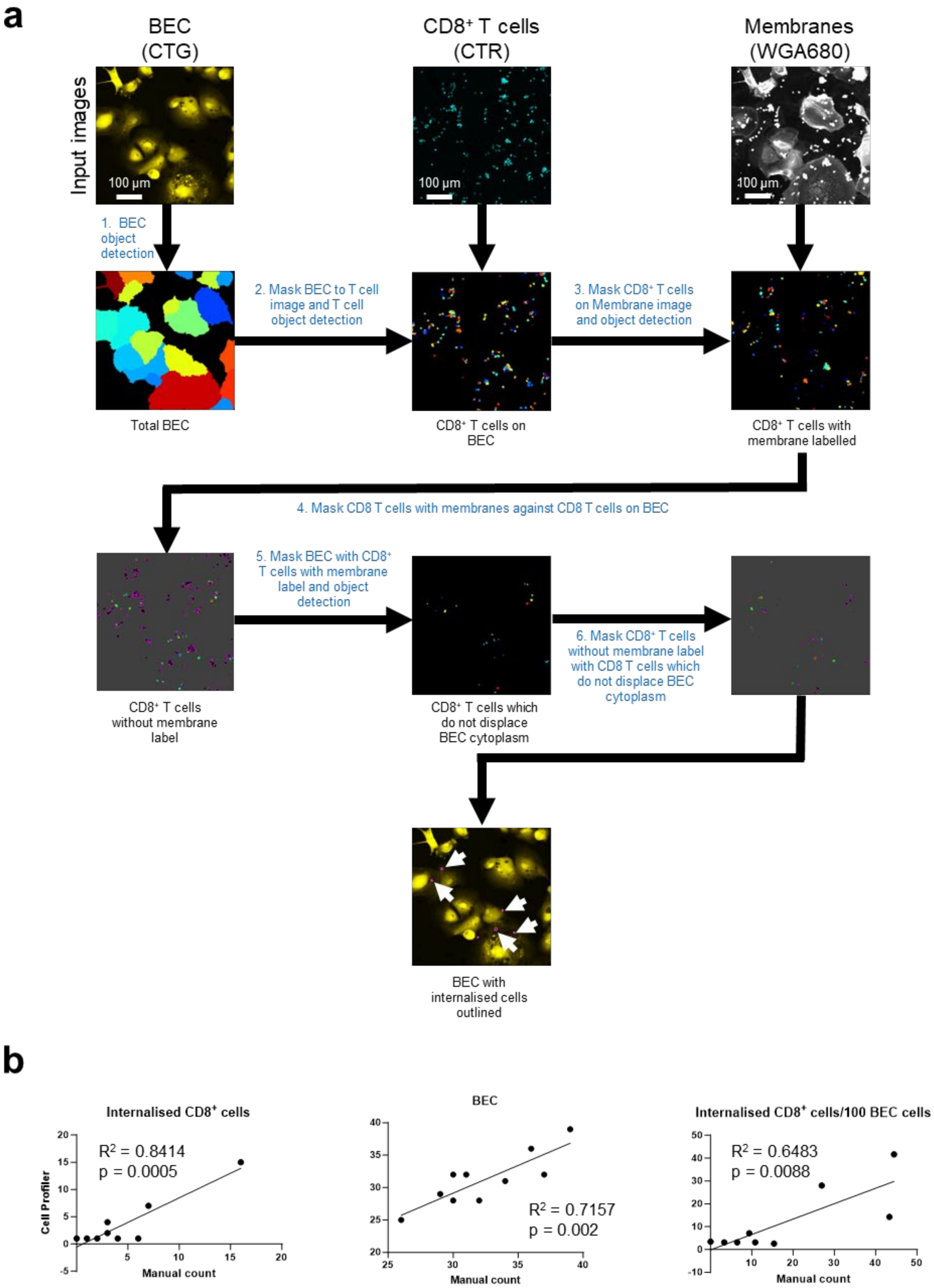

a
BEC (CTG)
CD8⁺ T cells (CTR)
Membranes (WGA680)
Input images
100 µm
1. BEC object detection
2. Mask BEC to T cell image and T cell object detection
3. Mask CD8⁺ T cells on Membrane image and object detection
Total BEC
CD8⁺ T cells on BEC
CD8⁺ T cells with membrane labelled
4. Mask CD8 T cells with membranes against CD8 T cells on BEC
5. Mask BEC with CD8⁺ T cells with membrane label and object detection
6. Mask CD8⁺ T cells without membrane label with CD8 T cells which do not displace BEC cytoplasm
CD8⁺ T cells without membrane label
CD8⁺ T cells which do not displace BEC cytoplasm
BEC with internalised cells outlined
b
Internalised CD8⁺ cells
Cell Profiler
Manual count
R² = 0.8414
p = 0.0005
BEC
R² = 0.7157
p = 0.002
Internalised CD8⁺ cells/100 BEC cells
R² = 0.6483
p = 0.0088

Figure 3. CellProfiler can be used to accurate quantify internalised CD8$^+$ T cells within BEC. A. Flow diagram showing an example of our bespoke CellProfiler-segregation and discovery pipeline for detecting internalised CD8$^+$ T cells within biliary epithelial cells (BEC). Numbers in the following description correspond to steps outlined in figure. (1) Objects were generated by detecting individual BEC (CellTracker™ Green; CTG, yellow) and (2) these were used to mask the matching T cell channel (CellTracker™ Red; CTR, cyan) to remove T cells not found in the same areas as BEC. (3) These T cells were then detected and used to mask the membrane label image (wheat germ agglutinin-Alexa Fluor 680; WGA680, grey). (4) T cells which possessed membrane labels (and therefore not internalised) were deleted from total T cell objects detected. (5) The new objects generated from the masking were then used to further mask the BEC channel. (6) Objects detected following this were T cells which possessed low membrane labelling but were not localised at areas of displayed BEC cytoplasm (displaced). Objects identified at this stage underwent further masking against objects pertaining to total CD8s without membrane labelling only. The resulting objects identified BEC cytoplasm-negative, membrane-label negative cells, which were classed as internalised for quantification. Pipeline file is supplied with this article as a supplementary software file. **B.** Linear regression analysis of manual cell counts versus CellProfiler analysis. Ten randomly chosen fields of view, of a 4 h BEC and CD8$^+$ T cell co-culture experiment, were used to optimise and validate the CellProfiler pipeline. Manual counts for total BEC, CD8$^+$ T cells and number of internalised T cells/100 BEC were plotted against those generated by CellProfiler and a linear regression was fitted. Values are plotted for each field of view.

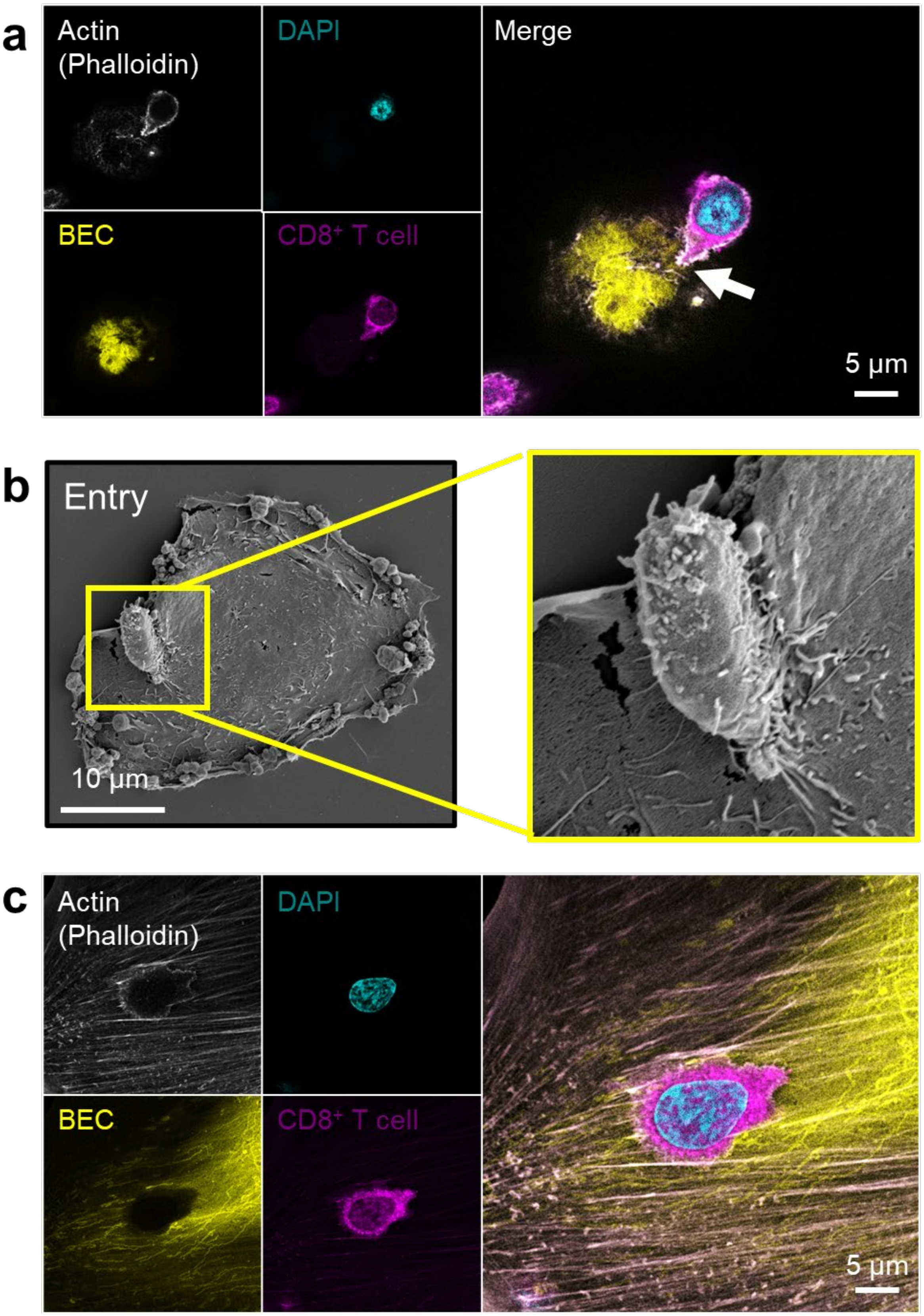
a
Actin
(Phalloidin)
DAPI
Merge
BEC
CD8+ T cell
5 µm
b
Entry
10 µm
c
Actin
(Phalloidin)
DAPI
BEC
CD8+ T cell
5 µm

Figure 4. Activated CD8$^+$ T cells undergo cytoskeletal rearrangements, form filopodia, and polarise towards the surface of the BEC. 48 h activated peripheral blood-derived CD8$^+$ T cells were co-cultured with biliary epithelial cells (BEC) for 4 h prior to undergoing specific preparation steps for individual imaging modalities. **A.** Multichannel super-resolution Airyscan confocal micrograph from Fig. 4B, of phalloidin-labelled (grey) co-cultured cells showing CD8$^+$ T cell (CellTracker™ Red; magenta) polarising towards a single BEC. Image displays the formation of actin-rich filopodia at the surface of BEC (CellTracker™ Green-labelled, yellow). BEC nucleus (DAPI; cyan) is not visible as image shows confocal plane only at the upper surface of the BEC. **B.** Scanning electron microscopy (SEM) image showing polarised CD8$^+$ T cell partially internalised into BEC. Inset (yellow box) shows the CD8$^+$ T cell breaching the surface of the BEC. **C.** Multichannel Airyscan super-resolution confocal micrograph of a CD8$^+$ T cell fully internalised within a BEC and its relationship with the BEC cytoskeleton.

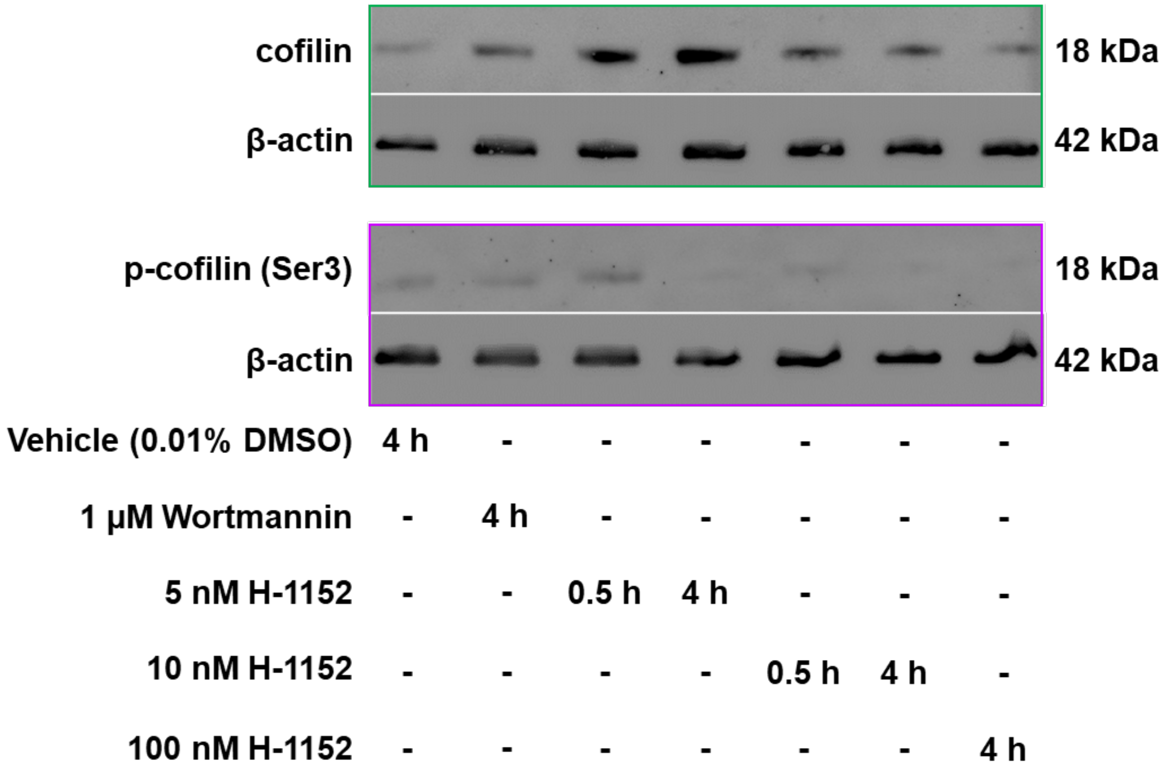

Figure 5. H-1152 treatment of CD8+ T cells prevented Ser3 phosphorylation of cofilin. Western blot comparing the presence of total cofilin to phosphorylated-cofilin from 48 h activated, blood-derived CD8+ T cells. Lysates were generated from T cells after treatment with DMSO (vehicle), wortmannin, or H-1152 (a selective ROCK inhibitor), at varying times and concentrations. Total cofilin and phosphorylated (Ser3) cofilin were ran on separate blots and β-actin was used as a loading control for each blot. – indicates absence of treatment.

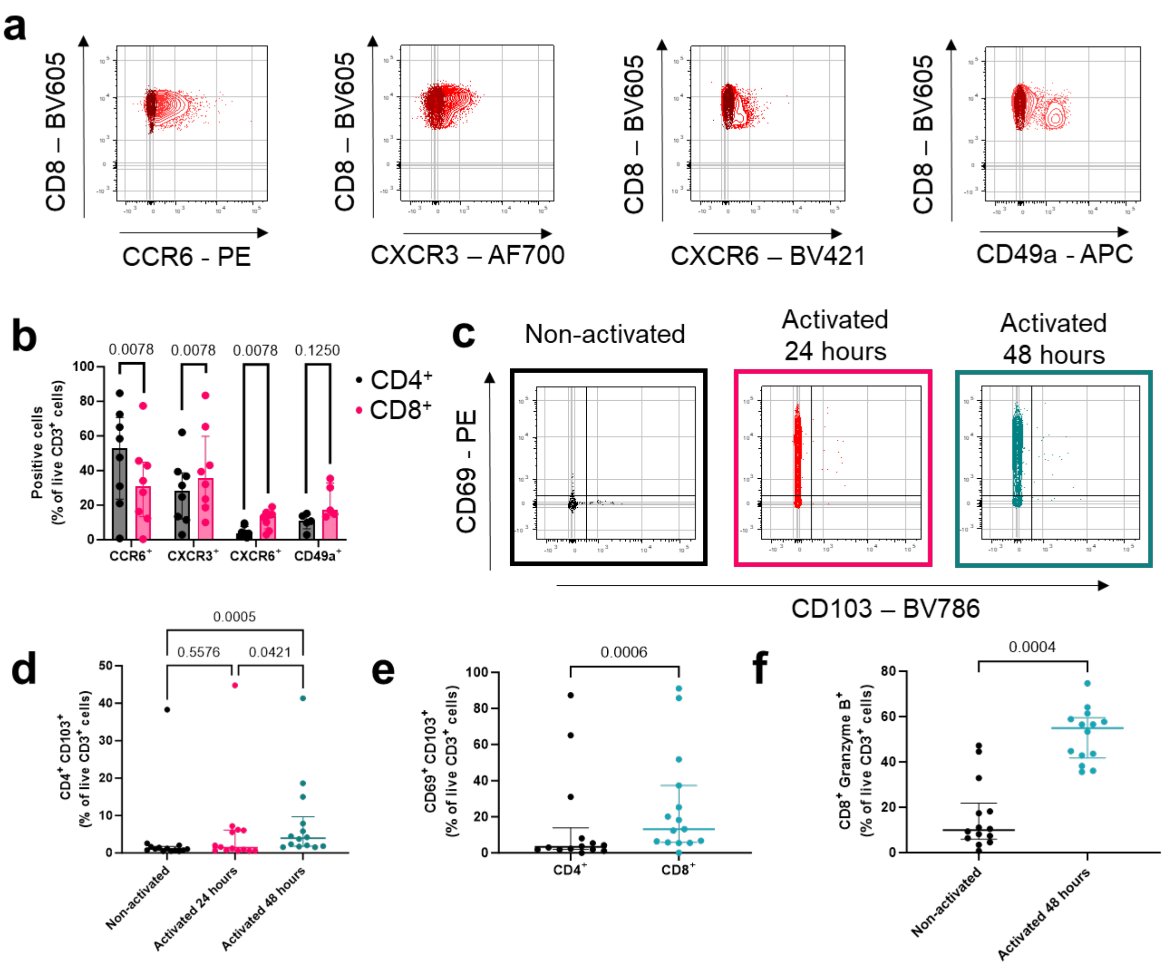
a
CD8 – BV605
CCR6 - PE
CXCR3 – AF700
CXCR6 – BV421
CD49a - APC
b
Positive cells (% of live CD3+ cells)
0.0078
0.0078
0.0078
0.1250
CD4+
CD8+
CCR6+
CXCR3+
CXCR6+
CD49a+
c
Non-activated
Activated 24 hours
Activated 48 hours
CD69 - PE
CD103 – BV786
d
CD4+ CD103+ (% of live CD3+ cells)
0.0005
0.5576
0.0421
Non-activated
Activated 24 hours
Activated 48 hours
e
CD69+ CD103+ (% of live CD3+ cells)
0.0006
CD4+
CD8+
f
CD8+ Granzyme B+ (% of live CD3+ cells)
0.0004
Non-activated
Activated 48 hours

Figure 6. Peripheral blood derived $CD8^+$ and $CD4^+$ T cells express biliary epithelium homing chemokine receptors, markers of residency, and cytolytic enzymes. Peripheral blood derived $CD8^+$ and $CD4^+$ T cells were phenotyped using flow cytometry specifically for the expression of receptors associated with recruitment, residency markers, and cytolytic enzymes. **A.** Representative contour plots (dark red) for CCR6 (n=8), CXCR3 (n=8), CXCR6 (n=8), and CD49a (n=5) expression by 24 h activated $CD8^+$ T cells. Black contours show isotype match control plots for each individual marker of interest. **B.** Percentage of $CD4^+$ and $CD8^+$ T cells expressing each cell surface marker shown in **A** 24 h post activation with α-CD3/CD28 stimulation. Values for individual patient samples are plotted. p values were generated using two-tailed Wilcoxon Signed-Rank tests. **C.** Representative contour plots showing expression of CD69 and CD103 by peripheral blood derived $CD4^+$ T cells in the absence of activation (black) or following 24 h (red) or 48 h (cyan) activation with α-CD3/CD28 stimulation. **D**. Percentage of $CD4^+$ T cells expressing CD103 (n=14). p values were generated using two-way Friedman tests. **E**. Comparison of percentage Granzyme B expression by $CD8^+$ T cells between non-activated cells and 48 hr post α-CD3/CD28 stimulation. p values were generated using two-tailed Wilcoxon Signed-Rank tests. (n=14). **F.** Comparison of the percentage of $CD69^+$ $CD103^+$ cells between $CD4^+$ and $CD8^+$ T cells 48 h post α-CD3/CD28 stimulation (n=15). p values were generated using two-tailed Wilcoxon Signed-Rank tests. p values are displayed in the figure for each statistical comparison made. n numbers represent the number of biologically independent patient samples.

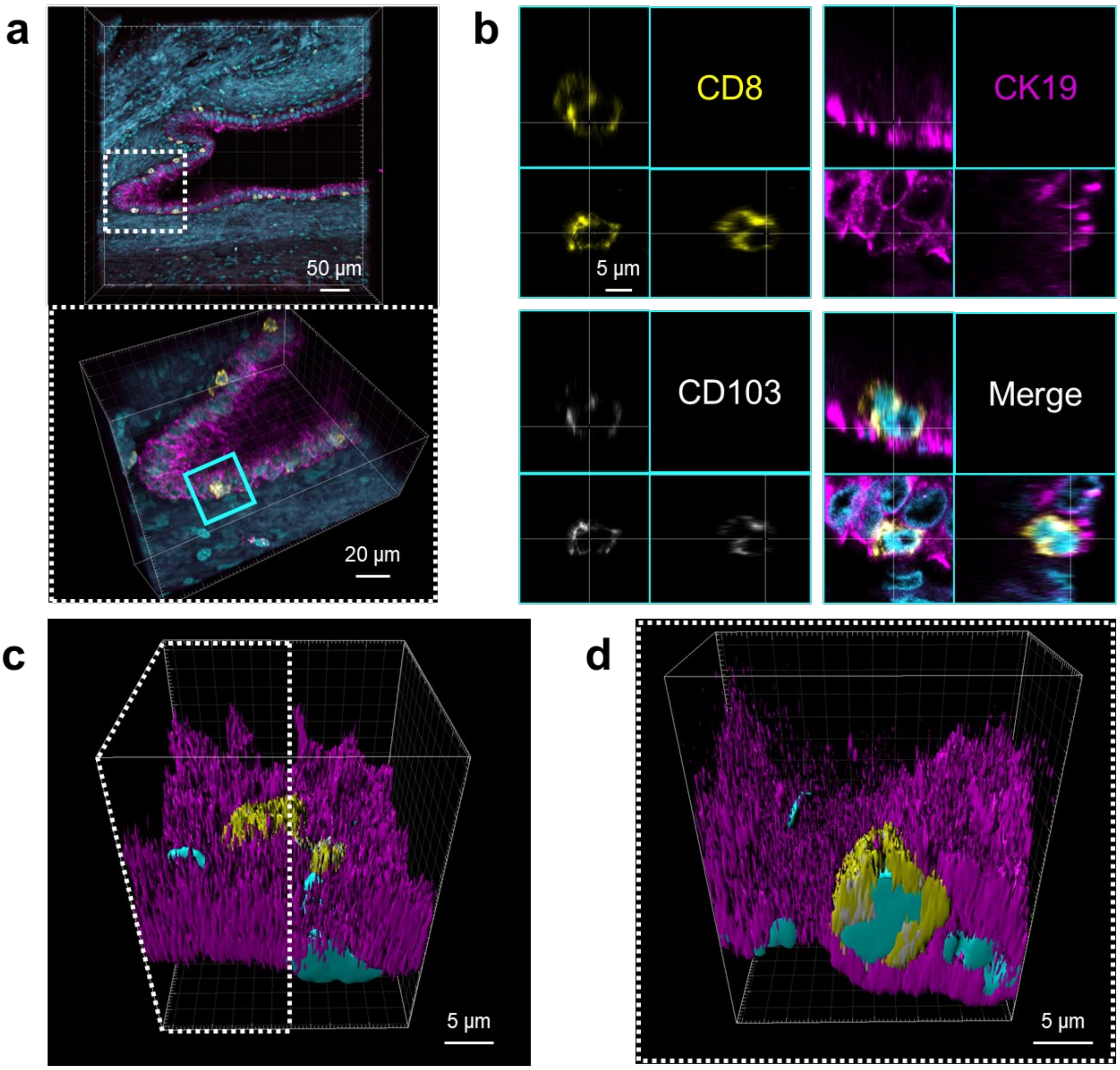
a
50 µm
20 µm
b
CD8
CK19
5 µm
CD103
Merge
c
5 µm
d
5 µm

Figure 7. **CD103$^+$ CD8$^+$ T cells are internalised within CK19$^+$ BEC in human PBC livers.** **A.** Immunohistochemistry (IHC) staining of a 50 μm-thick primary biliary cholangitis (PBC) liver tissue section showing cytokeratin-19$^+$ (CK19; magenta) bile ducts associated with CD103$^+$ (grey) CD8$^+$ (yellow) T cells. Top panel shows reconstructed Z-stack as viewed from above. Bottom panel shows angled view of inset from top panel (white dotted box). **B.** Orthographical, multichannel representations of **A** bottom panel inset (white box) demonstrating CD103$^+$ CD8$^+$ T cell with CK19 staining of biliary epithelial cells (BEC) localised around it. **C.** 3D-volume rendered representation of merged images shown in **B**. **D.** Cross-sectional view of panel **C** representing the area highlight by the dotted line. Nuclear staining using DAPI is shown in cyan.

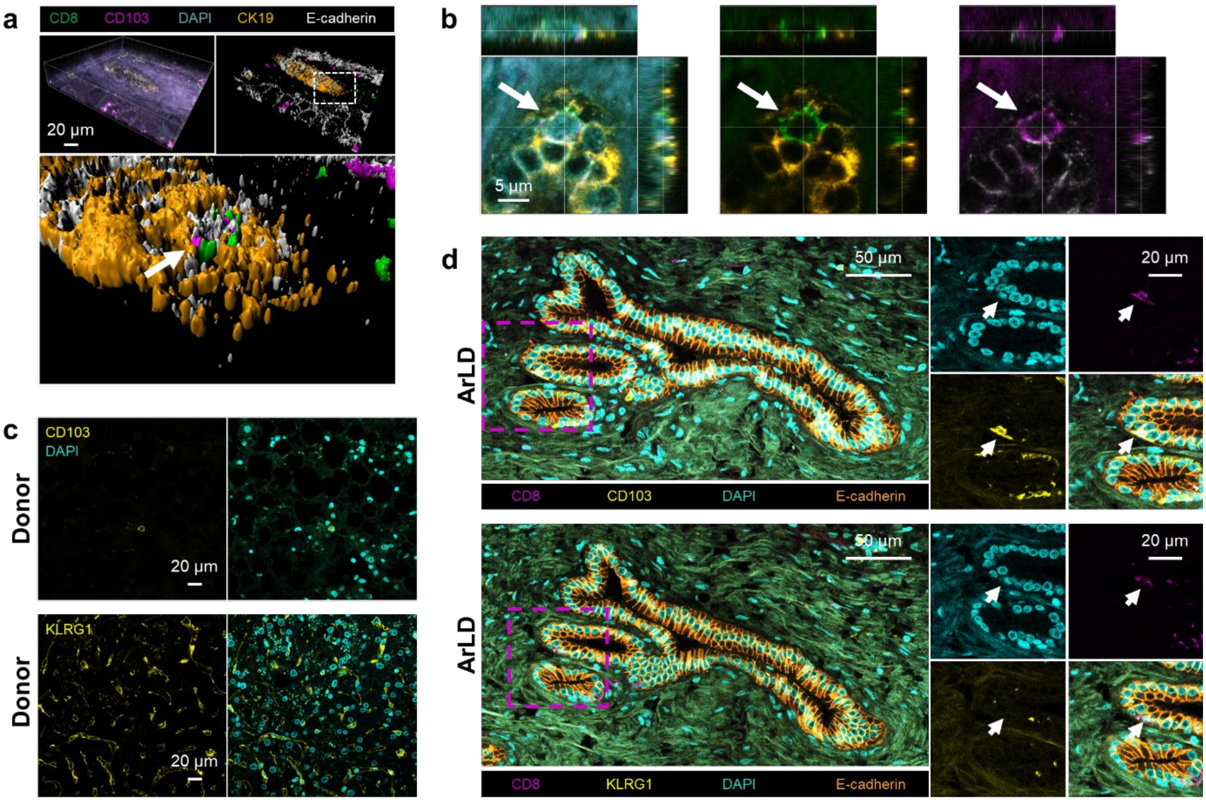
a
CD8
CD103
DAPI
CK19
E-cadherin
20 µm
b
5 µm
c
CD103
DAPI
Donor
20 µm
KLRG1
Donor
20 µm
d
ArLD
50 µm
20 µm
CD8
CD103
DAPI
E-cadherin
ArLD
50 µm
20 µm
CD8
KLRG1
DAPI
E-cadherin

Figure 8. **$CD8^+$ T cells internalised by BEC express CD103 but not KLRG1. A.** Immunohistochemistry (IHC) staining of a 50 µm-thick primary biliary cholangitis (PBC) liver tissue section showing a $CD103^+$ (magenta) $CD8^+$ (green) T cells adhered to E-cadherin$^+$ (grey) cytokeratin-19$^+$ (CK19; orange) biliary epithelial cells (BEC) which form the bile ducts. Top right panel shows 3D-volume rendered version of the left image. Bottom panel shows magnified image from of the top-right panel (white dotted box) demonstrating $CD103^+$ $CD8^+$ T cell, surrounded by E-cadherin and CK19 (white arrow). DAPI (cyan) was not rendered to enhance the visualisation of the internalised T cell. **B.** Orthographical, multichannel representations of magnified image of the part **A** bottom panel, showing the localisation of the $CD103^+$ $CD8^+$ T cell. **C+D.** Example confocal images used for IHC-based semi-quantitative analysis of the location of $CD103^+$/$KLRG1^+$ $CD8^+$ T cells in liver tissues from non-cirrhotic donor livers or patients with chronic liver diseases. **C.** Example confocal micrographs of liver parenchyma from non-cirrhotic donor liver livers stained by IHC for CD8 and either CD103 (top panel; yellow) or KLRG1 (bottom panel; yellow). **D.** Location-matched representative images of serial liver tissue sections from a patient with alcohol related liver disease (ArLD) stained for E-cadherin (orange), CD8 (magenta) and either CD103 or KLRG1 (yellow). Right panels show multichannel representations of left panel insets (purple dotted boxes). Arrows in upper panel show a $CD103^+$ $CD8^+$ T cell adhered to the surface of E-cadherin$^+$ BEC. Bottom panel shows that the same cells do not express KLRG1 (bottom right panel).

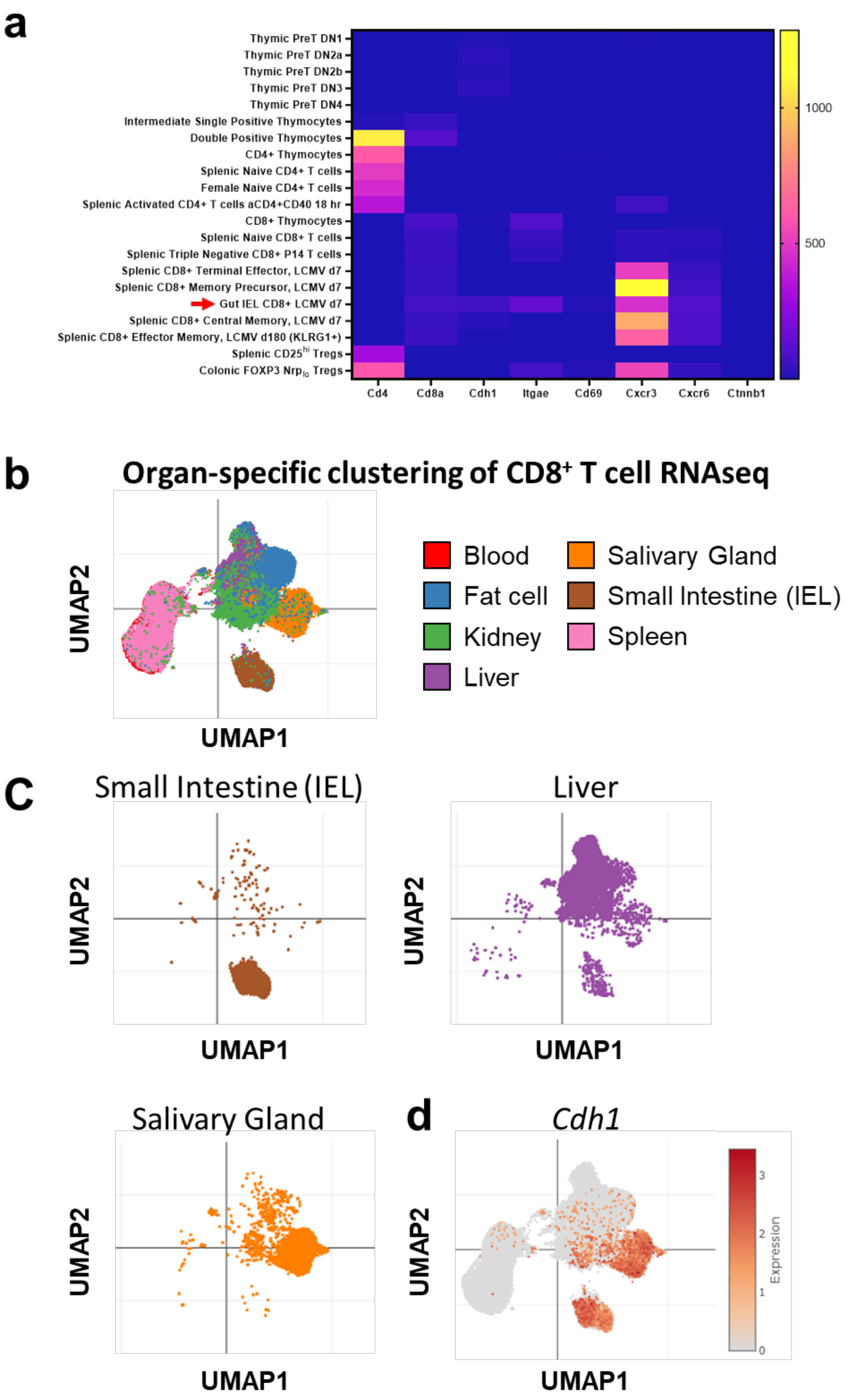
a
Thymic PreT DN1
Thymic PreT DN2a
Thymic PreT DN2b
Thymic PreT DN3
Thymic PreT DN4
Intermediate Single Positive Thymocytes
Double Positive Thymocytes
CD4+ Thymocytes
Splenic Naive CD4+ T cells
Female Naive CD4+ T cells
Splenic Activated CD4+ T cells aCD4+CD40 18 hr
CD8+ Thymocytes
Splenic Naive CD8+ T cells
Splenic Triple Negative CD8+ P14 T cells
Splenic CD8+ Terminal Effector, LCMV d7
Splenic CD8+ Memory Precursor, LCMV d7
Gut IEL CD8+ LCMV d7
Splenic CD8+ Central Memory, LCMV d7
Splenic CD8+ Effector Memory, LCMV d180 (KLRG1+)
Splenic CD25hi Tregs
Colonic FOXP3 Nrplo Tregs
Cd4
Cd8a
Cdh1
Itgae
Cd69
Cxcr3
Cxcr6
Ctnnb1
1000
500
b
Organ-specific clustering of CD8+ T cell RNAseq
UMAP2
UMAP1
Blood
Fat cell
Kidney
Liver
Salivary Gland
Small Intestine (IEL)
Spleen
c
Small Intestine (IEL)
Liver
Salivary Gland
d
Cdh1
Expression
0
1
2
3
UMAP2
UMAP1

Figure 9. E-cadherin$^+$ CD8$^+$ T cells are evolutionarily conserved in mice and enriched in liver, salivary glands, and gut intraepithelial lymphocyte populations. Open-source RNA sequencing (RNAseq) data was accessed via the Immunological Genome Project (ImmGen; https://www.immgen.org) and interrogated using their data browsing tools. **A.** Heatmap showing changes in gene expression of select genes across populations of murine early thymocytes, CD4$^+$ T cells, and CD8$^+$ T cells, as determined by ultra-low input RNA sequencing (ULI RNAseq). Changes in expression were normalised to median values per gene. Original data published in Mingueneau *et* al, 2013[4] (main reference 47). **B+C.** Gene clustering of scRNAseq data of resident CD8$^+$ populations from wild type C57BL/6 mice tissue, originally from Crowl *et al*, 2014[1] (main reference 48). UMAP plots are annotated based on organ ontology. **D.** Relative expression patterns of *Cdh1* across murine tissue resident CD8$^+$ T cell populations shown in **B**. Right heat scale shows changes in expression. All UMAP plots were generated using the Broad Single Cell Institute portal through the ImmGen website.

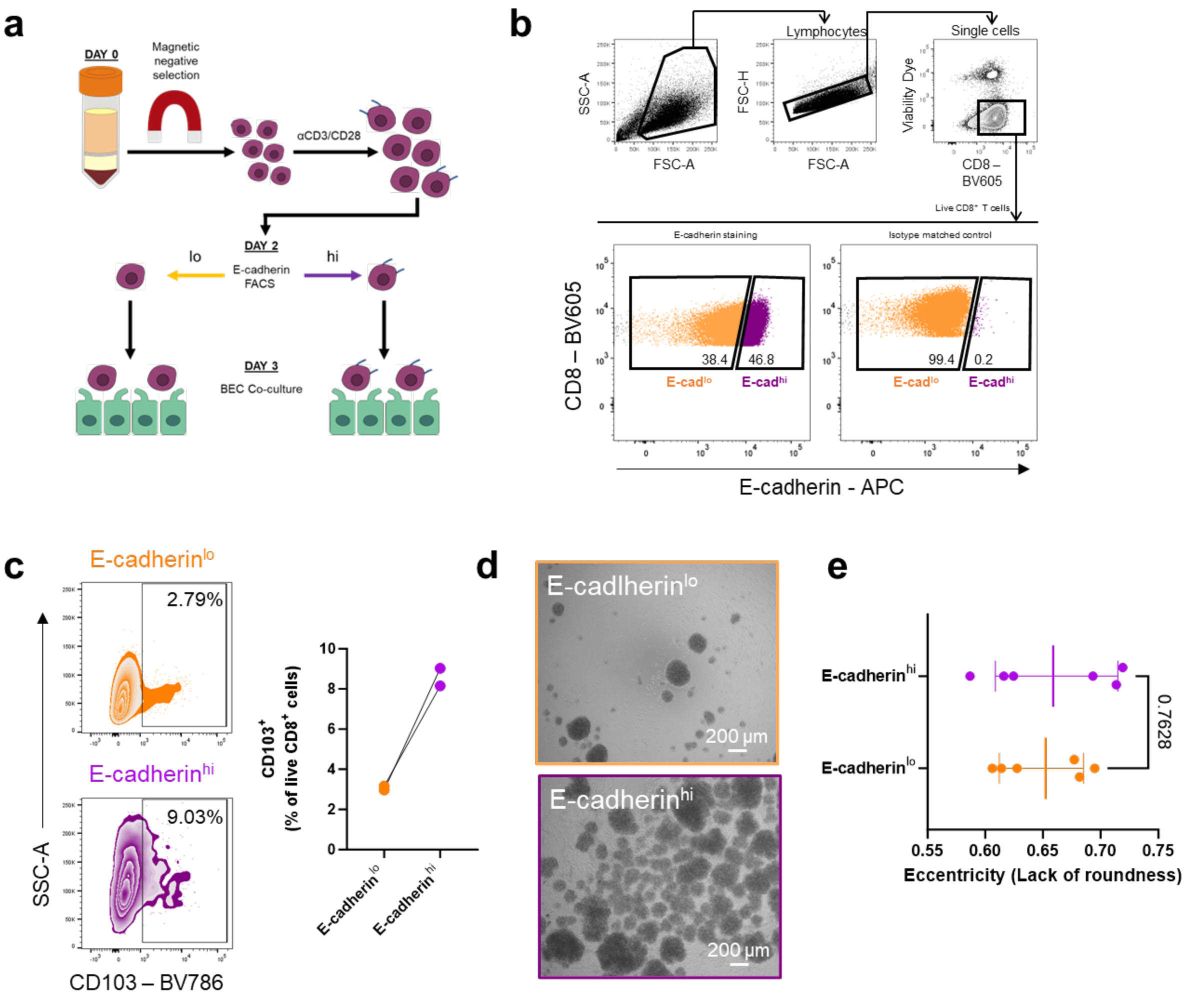
a
DAY 0
Magnetic negative selection
αCD3/CD28
DAY 2
lo
hi
E-cadherin FACS
DAY 3
BEC Co-culture
b
Lymphocytes
Single cells
SSC-A
FSC-A
FSC-H
FSC-A
Viability Dye
CD8 – BV605
Live CD8⁺ T cells
E-cadherin staining
Isotype matched control
CD8 – BV605
38.4
46.8
99.4
0.2
E-cadlo
E-cadhi
E-cadherin - APC
c
E-cadherinlo
2.79%
E-cadherinhi
9.03%
SSC-A
CD103 – BV786
CD103⁺ (% of live CD8⁺ cells)
E-cadherin lo
E-cadherin hi
d
E-cadlherinlo
200 µm
E-cadherinhi
200 µm
e
E-cadherinhi
E-cadherinlo
0.7628
0.55 0.60 0.65 0.70 0.75
Eccentricity (Lack of roundness)

Figure 10. E-cadherin^{hi} CD8^{+} T cells show higher proliferation, aggregation and CD103 expression than E-cadherin^{lo} CD8^{+} T cells. A. Graphical representation of the experiment pipeline. CD8^{+} T cells derived from healthy volunteer peripheral blood mononuclear cells (PBMCs), isolated using magnetic negative selection, were activated by α-CD3/CD28 stimulation and cultured for 48 h. E-cadherin^{hi} CD8^{+} T cells and E-cadherin^{lo} CD8^{+} T cells were then isolated using fluorescence activated cell sorting (FACS). T cells were then rested for 24 h, labelled with CellTracker™ Red, and co-cultured with CellTracker™ Green-labelled biliary epithelial cells (BEC) for 4 h. Cells were fixed and then imaged by fluorescence microscopy. **B.** Representative gating strategy for sorting of live CD8^{+} T cells E-cadherin^{hi} (purple) and E-cadherin^{lo} (orange) populations. **C.** Representative zebra plots of CD103^{+} CD8^{+} T cells in sorted E-cadherin^{hi} (purple) and E-cadherin^{lo} (orange) populations. n=2 biologically independent experiments. **D.** Phase contrast images of E-cadherin^{hi} CD8^{+} T cells (purple border) and E-cadherin^{lo} CD8^{+} T cells (orange border) 24 h after being sorted and seeded at equal concentrations. **E**. Quantification of eccentricity of internalised E-cadherin^{hi} and E-cadherin^{lo} CD8^{+} T cells. Nine fields of view were analysed from triplicate wells. Mean values/technical repeat are plotted. Error bars represent median and interquartile range. n=2 biologically independent experiments. Statistics were derived from an unpaired two-tailed Students t-test. T=0.3101, df=10. p values are displayed in the figure for each statistical comparison made.

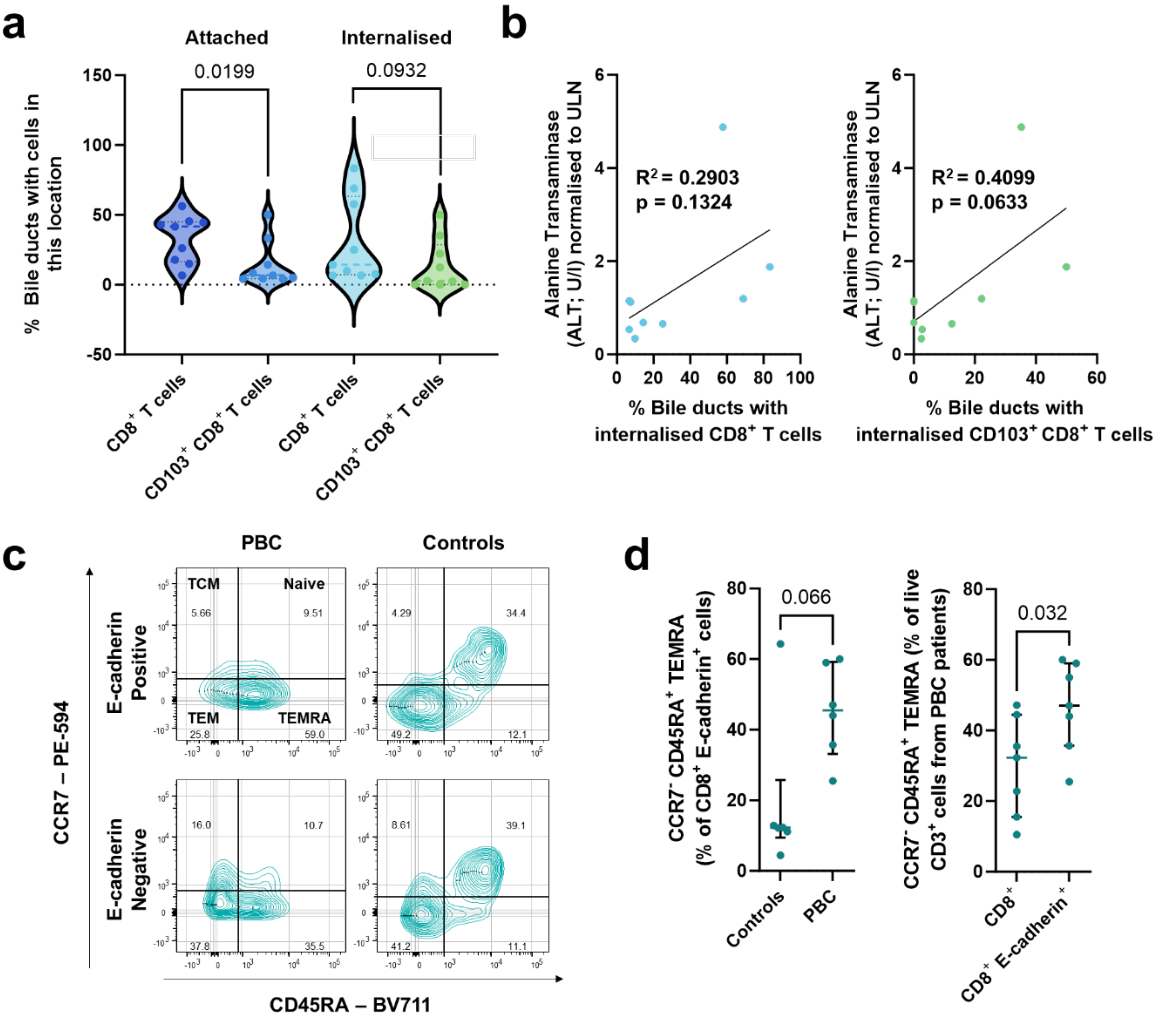

a
Attached
Internalised
0.0199
0.0932
% Bile ducts with cells in this location
CD8+ T cells
CD103+ CD8+ T cells
b
R2 = 0.2903
p = 0.1324
R2 = 0.4099
p = 0.0633
Alanine Transaminase (ALT; U/l) normalised to ULN
% Bile ducts with internalised CD8+ T cells
% Bile ducts with internalised CD103+ CD8+ T cells
c
PBC
Controls
TCM
Naive
TEM
TEMRA
E-cadherin Positive
E-cadherin Negative
CCR7 – PE-594
CD45RA – BV711
d
0.066
0.032
CCR7- CD45RA+ TEMRA (% of CD8+ E-cadherin+ cells)
CCR7- CD45RA+ TEMRA (% of live CD3+ cells from PBC patients)
Controls
PBC
CD8+
CD8+ E-cadherin+

Figure 11. $CD8^+$ T cells in PBC patients can be found internalised within BEC in liver biopsies and represent different sub-populations compared to non-PBC controls. A. Violin plots showing the percentage of bile ducts with attached or internalised $CD8^+$ or $CD103^+$ $CD8^+$ T cells. Liver biopsies taken from primary biliary cholangitis (PBC) patients with active disease were stained for CD8, CD103 and cytokeratin-19 (CK19) by immunohistochemistry (IHC). Bile ducts were identified using CK19 staining and the frequency of $CD8^+$ and $CD8^+$ $CD103^+$ T cells either within biliary epithelial cells (BEC) or attached to bile ducts was quantified. Statistics were derived from two-tailed Mann-Whitney tests. n=9 biologically independent patient samples. **B.** Linear regression of analysis comparing the percentage of bile ducts possessing internalised $CD8^+$ or $CD8^+CD103^+$ T cells observed in patient liver biopsies against patient-matched alanine transaminase levels (ALT; U/l) normalised to upper limit of normal (ULN). Dfn/Dfd = 1, 7. **C+D.** Peripheral blood mononuclear cells (PBMCs) from PBC patients and haemochromatosis (HFE) controls were phenotyped 48 h after activation via α-CD3/CD28 stimulation using flow cytometry. Expression of CD45RA and CCR7 was assessed to determine the subset of memory T cells that represent E-cadherin$^+$ $CD8^+$ T cells. **C.** Representative contour plots for CCR7 and CD45RA expression by E-cadherin$^+$ $CD8^+$ or E-cadherin$^-$ $CD8^+$ cells from PBC and HFE patients. TCM – T central memory. TEM T effector memory. TEMRA – T effector memory expressing CD45RA. **D.** *Left*: comparison of the percentage of E-cadherin$^+$ $CD8^+$ T cells expressing TEMRA markers between PBC patients and control patients. n=6 biologically independent patient samples/disease condition. p value was generated from a two-tailed Mann-Whitney test. *Right*: comparison of the percentage of total $CD8^+$ T cells and E-cadherin$^+$ $CD8^+$ T cells expressing TEMRA markers in PBC patients. n=7 biologically independent patient samples. p value was generated from a two-tailed Wilcoxon test. Values for individual patient samples are plotted. p values are displayed in the figure for each statistical comparison made.

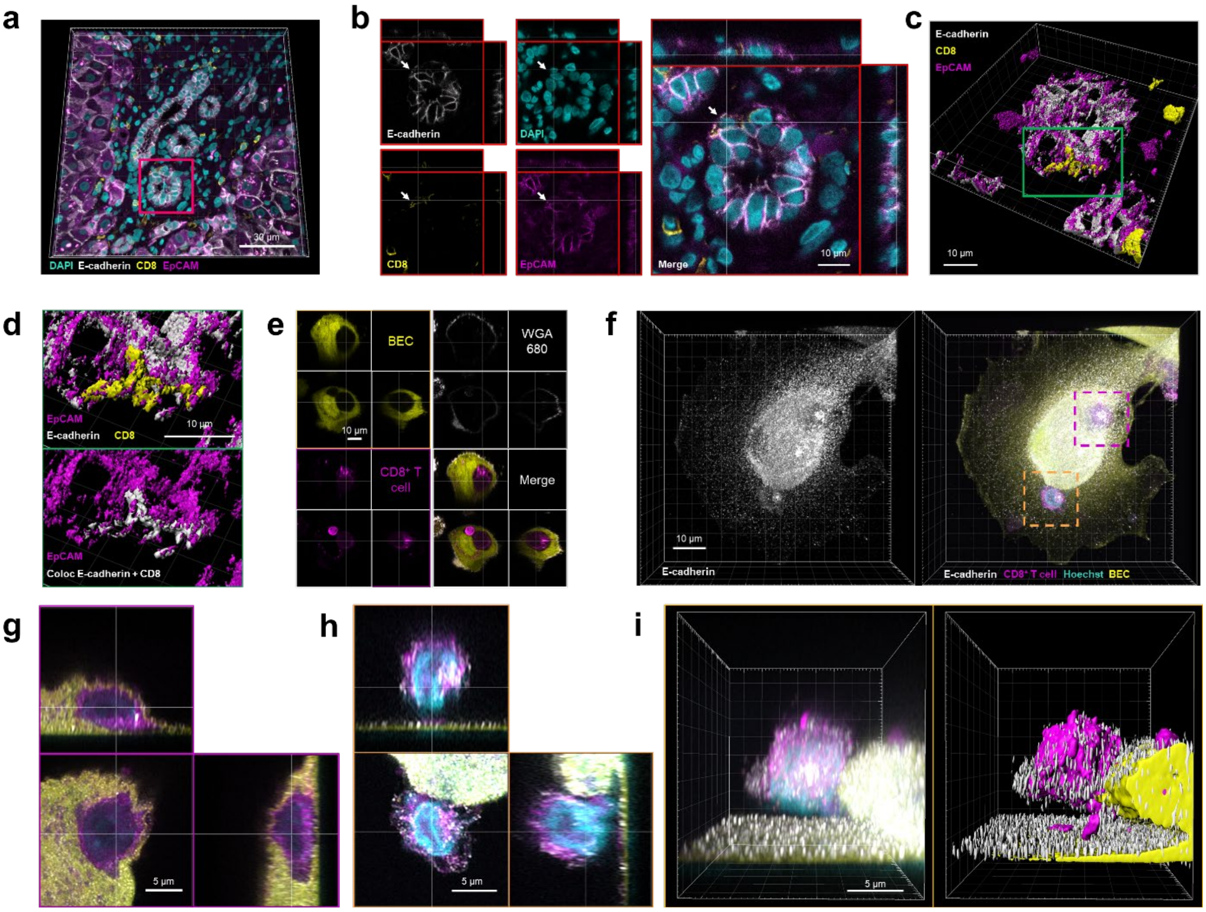

a
DAPI E-cadherin CD8 EpCAM
30 µm
b
E-cadherin
DAPI
CD8
EpCAM
Merge
10 µm
c
E-cadherin
CD8
EpCAM
10 µm
d
EpCAM
E-cadherin CD8
10 µm
EpCAM
Coloc E-cadherin + CD8
e
BEC
WGA 680
10 µm
CD8⁺ T cell
Merge
f
10 µm
E-cadherin
E-cadherin CD8⁺ T cell Hoechst BEC
g
5 µm
h
5 µm
i
5 µm

Figure 12. CD8$^+$ T cells from PBC patients can express E-cadherin when found within BEC in liver biopsies and when co-cultured with BEC *in vitro*. A. 3D-reconstruction of Z-stack confocal micrograph of PBC biopsy sample stained for CD8 (yellow), E-cadherin (white) and EpCAM (magenta); FOV is the same as Fig. 9A acquired by staining of a serial section. **B.** Multichannel orthographical images showing bile duct outlined in part **A** (red box) showing internalised CD8$^+$ T cell. FOV is the same as Fig. 9B. **C.** 3D-volume rendered reconstruction of panel **B. D**. Magnified inset images of panel **C** (green box). Bottom panel shows new channel comprised of colocalised CD8 and E-cadherin signal only (grey). **E.** Orthographical Airyscan confocal micrograph showing PBC patient-derived peripheral-blood CD8$^+$ T cells (CellTracker™ Red, magenta) internalised within a BEC (CellTracker™ Green; yellow) following detachment from the culture surface. Cells were labelled with Alexa Fluor 680-conjugated wheat germ agglutinin (WGA680; grey) after detachment with TrypLE™ Express, fixed, and then seeded to a glass bottom Ibidi µ-Slide VI 0.4 before imaging. **F**. 3D-reconstructed Airyscan Z-stack confocal micrographs showing co-cultured PBC blood-derived CD8$^+$ T cells (magenta) and BEC (yellow) stained for E-cadherin (grey) by immunocytochemistry (ICC). Cells were not permeabilised before staining. **G-H**. Orthographical representations of areas outlined in **F** showing an internalised T cell (**G**; magenta box) and an attached cell (**H**; orange box). **I.** 3D-reconstructed versions of **H**. Right panel shows 3D-volume rendered reconstruction.

SUPPLEMENTARY Tables

Supplementary table 1: Clinical parameters of PBC patient cohort from which peripheral blood samples were acquired for flow cytometry analysis and $CD8^{+}$ T cell isolation. All the patients were undergoing treatment with obeticholic acid (OCA). Cirrhosis was assessed by local pathologists and hepatologists according to national guidelines.

	**Cohort (n=7)**
**Age at time of sampling (mean, range)**	58±12
**Sex female n, (%)**	7(100)
**Concomitant autoimmune disease n, (%)**	3(42)
**Alkaline Phosphatase (ALP; mean range; IU/L)**	264±96
**Alanine Transaminase (ALT; mean, range; IU/L)**	31±19
**Cirrhosis n, (%)**	2(29)
**Ursodeoxycholic Acid (UDCA) responder n, (%)**	4(57)
**Second line treatment n, (%)**	3(43)

Supplementary table 2: Clinical parameters of PBC patient cohort from which biopsy samples were analysed. Cirrhosis, fibrosis stage, interface hepatitis, ductopenia and bile duct loss were assessed from liver biopsy samples by local pathologists and hepatologists according to national guidelines.

	**Cohort (n=9)**
**Age at time of biopsy (mean, range)**	51±15
**Sex male n (%)**	2 (23)
**Concomitant autoimmune disease n (%)**	4(44)
**Alkaline Phosphatase (ALP; median, range; IU/L)**	210 (139-234)
**Alanine Transaminase (ALT; median, range; IU/L)**	46 (27-49)
**Cirrhosis n, (%)**	1(12)
**Fibrosis:**	
**Grade 0,1,2 n, (%)**	7(77)
**Grade 3, 4 n, (%)**	2(23)
**Interface hepatitis n, (%)**	7(77)
**Ductopenia n, (%)**	1(11)
**Bile Duct loss n, (%)**	8(89)
**Ursodeoxycholic Acid (UDCA) treated n, (%)**	8(89)

Supplementary table 3: list of antibody details used for flow cytometry and fluorescence activated cell sorting staining within this investigation. For some antibodies, multiple lots were used throughout the course of this investigation. These are denoted as “multiple” in the lot number column. Antibodies are listed alphabetically by target. Isotype-matched controls (IMC) were used at matched concentrations to their experimental counterparts.

Target protein	Fluorochrome	Clone	Supplier	Catalogue number	Working dilution	Lot number
**β-catenin**	PE	15B8	Biolegend	862604	1:100	B303685
**CCR6**	PE	11A9	BD Biosciences	559562	1:50	7019800
**CCR7**	PE-594	150503	BD Biosciences	562381	1:50	2199957
**CD103**	BV786	Ber-ACT8	BD Biosciences	743654	1:50	2250662
**CD107a**	PE-Cy7	H4A3	Biolegend	328618	1:400	B284259
**CD127**	BV711	A019D5	Biolegend	351328	1:100	B393047
**CD161**	APC	HP-3G10	Biolegend	339912	1:100	B302178
**CD25**	BV421	2A3	BD Biosciences	564003	1:200	2206936
**CD27**	BUV395	L128	BD Biosciences	563815	1:100	7004623
**CD28**	BV650	CD28.2	Biolegend	302946	1:100	B315410
**CD3**	BV510	OKT3	Biolegend	317332	1:100	Multiple
**CD38**	PE-Cy7	HIT2	eBioscience	25038942	1:400	2255543
**CD4**	BUV395	RPA-T4	BD Biosciences	564724	1:400	Multiple
**CD45RA**	BV711	HI100	Biolegend	304130	1:400	B345247
**CD49a**	APC	TS2/7	Biolegend	328314	1:100	B270613
**CD57**	PerCP	HNK-1	Biolegend	359622	1:400	B321641
**CD69**	PE	FN50	Biolegend	310906	1:100	B352653
**CD8**	BV605	SK1	Biolegend	344742	1:400	Multiple
**CXCR3**	AF700	G025H7	Biolegend	353742	1:33	B330851
**CXCR6**	BV421	K041E5	Biolegend	356014	1:100	Multiple
**E-cadherin**	APC	67A4	Biolegend	324108	1:25	Multiple
**Granzyme B**	PerCPCy5.5	QA16A02	Biolegend	372212	1:100	B357972
**IMC (mouse IgG1)**	APC	MOPC-21	Biolegend	400120	-	Multiple
**KLRG1**	FITC	SA231A2	Biolegend	367714	1:100	B320897
**LFA1**	AF700	m24	Biolegend	363422	1:200	B283404
**Perforin**	PerCPCy5.5	dG9	Biolegend	308114	1:50	B282783

Supplementary table 4: list of antibody details used for immunohistochemistry staining within this investigation. For some antibodies, multiple lots were used throughout the course of this investigation. These are denoted as “multiple” in the lot number column. Isotype-matched control antibodies were used at a matched concentration to the antibody for which they were the control. Antibodies are listed alphabetically by target. Isotype-matched controls (IMC) were used at matched concentrations to their experimental counterparts.

Target protein	Fluorochrome	Clone	Supplier	Catalogue number	Working dilution	Lot number
**β-catenin**	Unconjugated	12F7	Biolegend	844602	1:50	B305601
**CD103**	Unconjugated	EPR22590-27	Abcam	ab224202	1:500	Multiple
**CD3**	Unconjugated	F7.2.38	Abcam	ab17143	1:25	GR3395244-2
**CD4**	Unconjugated	OTI5D9	Novus Biologicals	NBP2-46149	1:50	Multiple
**CD69**	Unconjugated	8B6	Fisher Scientific/Invitrogen	MA5-15612	1:50	WI3371233
**CD8**	Unconjugated	4B11	Fisher Scientific/Invitrogen	MA1-80231	1:50	Multiple
**Cytokeratin-19**	Unconjugated	EP1580Y	Abcam	ab52625	1:2000	GR3255534-19
**Cytokeratin-19**	Unconjugated	1H6	Fisher Scientific/Invitrogen	MA5-15862	1:1000	YH4011082A
**E-cadherin**	Unconjugated	36/E-Cadherin	BD Biosciences	610181	1:50	Multiple
**EpCAM**	Unconjugated	EGP40/1372	Abcam	ab218448	1:200	GR3417029-2
**IMC (Mouse IgG1)**	Unconjugated	MOPC-21	Biolegend	400166	-	B317345
**IMC (Mouse IgG2a)**	Unconjugated	MG2a-53	Biolegend	401508	-	B304626
**IMC (Mouse IgG2b)**	Unconjugated	MG2b-57	Biolegend	401216	-	B297109
**IMC (Rabbit)**	Unconjugated	DA1E	Cell Signalling Tech.	3900S	-	50
**KLRG1**	Unconjugated	2388C	R&D Systems	70293	1:50	CLPU0221081
**Mouse IgG1**	AF488	Polyclonal	Fisher Scientific/Invitrogen	A-21121	1:1000	2339820
**Mouse IgG1**	AF647	Polyclonal	Fisher Scientific/Invitrogen	A-21240	1:1000	2652975
**Mouse IgG2a**	AF546	Polyclonal	Fisher Scientific/Invitrogen	A-21133	1:1000	2447873
**Mouse IgG2b**	AF647	Polyclonal	Fisher Scientific/Invitrogen	A-21242	1:1000	2273692
**Rabbit IgG**	AF647 Plus	Polyclonal	Fisher Scientific/Invitrogen	A32733	1:1000	WL333739
**Rabbit IgG**	Dylight 594	Polyclonal	Vector Laboratories	DI-1094-1.5	1:500	ZH0527
**Rabbit IgG**	Dylight 488	Polyclonal	Vector Laboratories	DI-1088-1.5	1:500	ZH0423

Supplementary table 5: list of antibody details used for immunocytochemistry staining within this investigation. For some antibodies, multiple lots were used throughout the course of this investigation. These are denoted as "multiple" in the lot number column. Isotype-matched control antibodies were used at a matched concentration to the antibody for which they were the control. Antibodies are listed alphabetically by target. Isotype-matched controls (IMC) were used at matched concentrations to their experimental counterparts.

Target protein	Fluorochrome	Clone	Supplier	Catalogue number	Working dilution	Lot number
**α-tubulin**	Unconjugated	TU-01	Fisher Scientific/Invitrogen	MA1-19162	1:50	543170
**β-catenin**	Unconjugated	12F7	Biolegend	844602	1:50	Multiple
**Cytokeratin-19**	Unconjugated	EP1580Y	Abcam	ab52625	1:1000	GR3255534-19
**E-cadherin**	Unconjugated	36/E-Cadherin	BD Biosciences	610181	1:50	Multiple
**EpCAM**	Unconjugated	HEA-125	Progen Biotechnik	61004	1:50 (from 0.5 mg/ml)	703131B
**IMC (Mouse IgG1)**	Unconjugated	MOPC-21	Biolegend	400166	-	B317345
**IMC (Mouse IgG2a)**	Unconjugated	MG2a-53	Biolegend	401508	-	B304626
**IMC (Rabbit)**	Unconjugated	DA1E	Cell Signalling Tech.	3900S	-	50
**Mouse IgG1**	AF488	Polyclonal	Fisher Scientific/Invitrogen	A-21121	1:1000	2339820
**Mouse IgG1**	AF647	Polyclonal	Fisher Scientific/Invitrogen	A-21240	1:1000	2652975
**Mouse IgG2a**	AF546	Polyclonal	Fisher Scientific/Invitrogen	A-21133	1:1000	2447873
**Rabbit IgG**	AF647 Plus	Polyclonal	Fisher Scientific/Invitrogen	A32733	1:1000	WL333739

Supplementary table 6: list of antibody details used for purposes within this investigation other than cell and tissue immunostaining. Antibodies are listed alphabetically by target and their purpose is shown on the end column.

Target protein	Fluorochrome	Clone	Supplier	Catalogue Number	Working dilution	Lot Number	Purpose
**Cofilin**	Unconjugated	GT567	Fisher Scientific/Invitrogen	MA5-17275	1:500	YG3988356	Western blotting
**Phospho-cofilin (Ser3)**	Unconjugated	Polyclonal	Fisher Scientific/Invitrogen	44-1072G	1:500	2456324	Western blotting
**β-actin**	Unconjugated	AC-15	Merck	A5441	1:2000	0000126949	Western blotting
**Rabbit IgG**	AF488	Polyclonal	Fisher Scientific/Invitrogen	A-11008	1:500	1981125	Western blotting
**Mouse IgGa**	AF488	Polyclonal	Fisher Scientific/Invitrogen	A-21131	1:500	2273777	Western blotting
**Mouse IgG1**	AF647	Polyclonal	Fisher Scientific/Invitrogen	A-21240	1:500	2652974	Western blotting
**CD103**	Unconjugated	EPR22590-27	Abcam	ab224202	1:50	GR3309850-5	Functional Blockade

SUPPLEMENTARY REFERENCES

1 Crowl, J. T. *et al.* Tissue-resident memory CD8(+) T cells possess unique transcriptional, epigenetic and functional adaptations to different tissue environments. *Nature immunology* **23**, 1121-1131 (2022). https://doi.org:10.1038/s41590-022-01229-8

2 Hofmann, M. & Pircher, H. E-cadherin promotes accumulation of a unique memory CD8 T-cell population in murine salivary glands. *Proceedings of the National Academy of Sciences of the United States of America* **108**, 16741-16746 (2011). https://doi.org:10.1073/pnas.1107200108

3 Huang, B. *et al.* NUDT1 promotes the accumulation and longevity of CD103(+) T(RM) cells in primary biliary cholangitis. *Journal of hepatology* **77**, 1311-1324 (2022). https://doi.org:10.1016/j.jhep.2022.06.014

4 Mingueneau, M. *et al.* The transcriptional landscape of alphabeta T cell differentiation. *Nature immunology* **14**, 619-632 (2013). https://doi.org:10.1038/ni.2590
