# Supplementary material for: Expression of E-cadherin by CD8+ T cells promotes their invasion into biliary epithelial cells
Source: Nat Commun. 2024 Jan 29;15:853. doi: 10.1038/s41467-024-44910-2 (PMC10825166; doi:10.1038/s41467-024-44910-2)
Supplement: Supplementary file 3 — Reporting Summary [file 41467_2024_44910_MOESM3_ESM.pdf]

nature portfolio

Corresponding author(s): Dr Scott Philip Davies, Prof Ye Htun Oo

Last updated by author(s): Dec 13, 2023

Reporting Summary

Nature Portfolio wishes to improve the reproducibility of the work that we publish. This form provides structure for consistency and transparency in reporting. For further information on Nature Portfolio policies, see our Editorial Policies and the Editorial Policy Checklist.

Statistics

For all statistical analyses, confirm that the following items are present in the figure legend, table legend, main text, or Methods section.

n/a	Confirmed	
☐	☒	The exact sample size (*n*) for each experimental group/condition, given as a discrete number and unit of measurement
☐	☒	A statement on whether measurements were taken from distinct samples or whether the same sample was measured repeatedly
☐	☒	The statistical test(s) used AND whether they are one- or two-sided<br>*Only common tests should be described solely by name; describe more complex techniques in the Methods section.*
☐	☒	A description of all covariates tested
☐	☒	A description of any assumptions or corrections, such as tests of normality and adjustment for multiple comparisons
☐	☒	A full description of the statistical parameters including central tendency (e.g. means) or other basic estimates (e.g. regression coefficient) AND variation (e.g. standard deviation) or associated estimates of uncertainty (e.g. confidence intervals)
☐	☒	For null hypothesis testing, the test statistic (e.g. *F*, *t*, *r*) with confidence intervals, effect sizes, degrees of freedom and *P* value noted<br>*Give P values as exact values whenever suitable.*
☒	☐	For Bayesian analysis, information on the choice of priors and Markov chain Monte Carlo settings
☒	☐	For hierarchical and complex designs, identification of the appropriate level for tests and full reporting of outcomes
☒	☐	Estimates of effect sizes (e.g. Cohen's *d*, Pearson's *r*), indicating how they were calculated

Our web collection on statistics for biologists contains articles on many of the points above.

Software and code

Policy information about availability of computer code

Data collection	The Gene Skyline data browser was used to access open source murine ULI RNA seq data obtained from murine thymocyte populations (http://rstats.immgen.org/Skyline/skyline.html). Single-cell RNAseq online databrowsers provided by lmmGem Single Cell Portal were used to interrogate open source single cell RNAseq data of murine CD8+ resident T cell populations. Fluorescence microscopes used throughout the report were operated using Zen v3.5 (black or blue edition; Carl Zeiss). Election microscopy images were acquired using software associated with the instruments used. Flow cytometry and fluorescence activated cell sorting (FACS) data was acquired using FACSDiva (BD Biosciences).
Data analysis	Analysis of co-cultured cells for quantifying internalized cells and lysosome association was performed was using a custom-made pipelines developed using CellProfiler v4.2.1 and v4.1.3. The cellprofiler pipeline is available at https://cellprofiler.org/published-pipelines. We will update the data availability statement to include all links to find the pipeline. CellProfiler will be informed of the new articles DOI to ensure the pipeline is linked to this article (as well as the associated pre-print). All flow cytometry data was analyzed using FlowJo vl0.8.1. Fluorescence microscopy images were generated using Zen v3.5 (blue edition; Carl Zeiss) or IMARIS for cell biologists v8.3 (Bitplane). New colocalisation channels were generated using IMARIS. Pixel intensity of confocal micrographs was analysed using Zen v3.5 (blue edition; Carl Zeiss). All data generated from these softwares were analyzed further using Graphpad Prism v9.5.1 or v10.1.1.

For manuscripts utilizing custom algorithms or software that are central to the research but not yet described in published literature, software must be made available to editors and reviewers. We strongly encourage code deposition in a community repository (e.g. GitHub). See the Nature Portfolio guidelines for submitting code & software for further information.

Data

Policy information about availability of data

All manuscripts must include a data availability statement. This statement should provide the following information, where applicable:

- Accession codes, unique identifiers, or web links for publicly available datasets
- A description of any restrictions on data availability
- For clinical datasets or third party data, please ensure that the statement adheres to our policy

Source raw data for all graphs within this paper (excluding flow cytometry contour plots) as well as raw western blot data, are provided within the source data file which has been published alongside this article. All other data associated with the article is available upon request. Aggregated data for the cohort of patients included in the study are provided in the supplementary tables.

This paper generated figures using data available from the Immunological Genome project (ImmGen; www.immgen.org). This included ultra-low input (ULI) RNAseq profiles of murine αβ CD4+ and CD8+ T cells from immune system human cell atlas which was browsed using the ImmGen RNAseq skyline portal:

(http://immunecellatlas.net/ICA_Skyline.php?gene=TP53&celltype=all&organ=all&datatype=rnaseq&scale=Local).

Single cell RNAseq data of murine tissue-resident CD8+ T cells was also accessed through ImmGen using Broad Institute data browsers:

(https://singlecell.broadinstitute.org/single_cell/study/SCP1865/murine-cd8-tissue-resident-t-cells?scpbr=immunological-genome-project#/)

Research involving human participants, their data, or biological material

Policy information about studies with human participants or human data. See also policy information about sex, gender (identity/presentation), and sexual orientation and race, ethnicity and racism.

Reporting on sex and gender

The sex and gender related to the individuals providing biological and clinical samples for our study was not collected. Only the sex ratio of sample populations were noted.

Reporting on race, ethnicity, or other socially relevant groupings

Race, ethnicity or any other socially relevant grouping information related to the individuals providing biological and clinical samples for our study was not collected.

Population characteristics

Live human subjects were not used in our study. Paraffin-embedded tissues were obtained from the bio bank associated with our study. These are formalin-fixed paraffin-embedded (FFPE) explanted liver tissues from consented patients with chronic disease liver disease which had undertaken a liver transplant. Non-cirrhotic donor liver tissues which were rejected for transplantation were used as controls.Fresh ex vivo tissue was also collected under the same study to isolate primary biliary epithelial cells (BEC). Clinical data relating to covariate characteristics of patients whom donated material was not collected nor referred to when selecting FFPE and fresh tissues. Disease of origin was also not considered when isolating BEC as they were passaged to a point where the influence of the disease would be lost. Clinical data relating to individuals from which non-cirrhotic donor liver tissues were sourced is not available to our institution.

FFPE biopsy samples from nine patients with ongoing primary biliary cholangitis (PBC), taken at the time of diagnosis, were also used in our study. Samples were selected based on tissue availability, lack of additional liver disease and if patients had active disease at the time of inquiry. Patients were 48 +/ years old, 11% female, and treatment-naive at the time of biopsy. 44% also possessed additional, non-liver autoimmune disease.

Blood was taken from healthy donors as well as patients with diagnosed ongoing primary biliary cholangitis, or haemchromatosis (HFE; iron overload). Healthy donors were bled from individuals based on their availability within the laboratories. Patients were bled upon visiting the clinics of Pro Ye Htun Oo and Dr Vincenzo Ronca. No other population characteristics were considered. Blood was taken from 7 patients with PBC. All of them were female and the mean age was 58±12 (mean ± SD). These features are in keeping with the general epidemiology of the disease. 7 controls were randomly selected from the HFE venisection clinic. These individuals undergo venesection as preventive measure for iron accumulation and do not have liver disease. We did not collect sex and gender data on this patient as not relevant for the study.

Recruitment

Human research participants in our study are consented for the provision of biological material with informed consent from within the liver clinics at the QE Hospital Birmingham. Tissue specimens from our bio bank were selected based on a their chronic liver disease diagnose at their time of transplantation. No other parameters were used. Non-cirrhotic donors have no clinic data attached to them. Healthy blood donors are bled after giving consent under the same study ethics, as and when the experiments were planned. Blood donors with active liver disease (PBC or HFE) were bled during their clinical appointments. Anonymity designations were matched to hospital identifying numbers to ensure the same patients were not used twice throughout experimental procedures.

Blood were collected from sequential patients in the dedicated PBC clinic. We excluded any individual with chronic liver disease other than PBC. Biopsies selection were randomly selected in the pool of patients with PBC underwent liver biopsy. However, since liver biopsy is not a common clinical practice in PBC (not indicated in the clinical guidelines for the diagnosis), the indication for the biopsy could have been a source of selection bias. However, the clinical and biochemical profile of the included patients are in keeping with the epidemiology of PBC in UK and, they are representative of randomly selected PBC cohort to the best of our knowledge.

Ethics oversight

Ethics for the collection of tissue and blood from patients with chronic liver disease, as well as the collection of peripheral blood from healthy volunteers was approved by the Local Research Ethics Committee (LREC) at the University of Birmingham under the study name: Inflammation in the development and outcome of liver disease. Integrated Research Application System (IRAS) number: 223072. Sponsor Reference number: RG_l7-256. REC reference number: 18-WA-0214.

Biopsy samples were provided by the QE Hospital Birmingham through an agreement with the Human Biomaterial Resource Centre (HBRC) at the University of Birmingham (UoB-ATF-POL-0002). Ethical approval for HBRC tissue collection was provided by REC Committee North West- Haydock; Ref 20/NW/0001. Sponsor Reference number: PKG: 2307-4364. Req 1237. PR: 2341
Ethics for the collection of tissue and blood from patients with chronic liver disease, as well as the collection of peripheral blood from healthy volunteers was approved by the Local Research Ethics Committee (LREC) at the University of Birmingham under the study name: Inflammation in the development and outcome of liver disease. Integrated Research Application System (IRAS) number: 223072. Sponsor Reference number: RG_l7-256. REC reference number: 18-WA-0214.

Note that full information on the approval of the study protocol must also be provided in the manuscript.

Field-specific reporting

Please select the one below that is the best fit for your research. If you are not sure, read the appropriate sections before making your selection.

☒ Life sciences ☐ Behavioural & social sciences ☐ Ecological, evolutionary & environmental sciences

For a reference copy of the document with all sections, see nature.com/documents/nr-reporting-summary-flat.pdf

Life sciences study design

All studies must disclose on these points even when the disclosure is negative.

Sample size

For semi-quantitative analysis of CD8+ T cell localization in patients with different forms of chronic liver disease (Figure 4B+C), six patients were analyzed per condition. This was previously sufficient to establish a pattern of localization of specific lymphocyte populations in our previous investigations (Wiggins et al, 2022).

Flow cytometry phenotyping of human peripheral blood mononuclear cells was performed for a minimum of 10 patients per disease condition as our previous reports demonstrated that this number was necessary to make both statistically and biological conclusions from our phenotyping. (Wiggins et al, Gut, 2022).

For other experiments, this decision for sample sizes was pragmatically operated on the basis of the observed biological variability and on the availability of samples in our cohort.

Data exclusions

For co-culture experiments, values obtained from automated analysis relating to images in which cells were obscured by artifacts (hairs or dying cells, for example) were excluded from final analyses. No other data was excluded.

Replication

For experiments involving co-cultures between biliary epithelial cells (BEC) and CD8+ T cells, a minimum of three experimental repeats were performed as this number was previously sufficient to achieve statistically significant differences when performing experimental interventions of cell-in-cell structure processes (Davies et al, Cell Reports, 2019). The exceptions are as follows:

Figure 1D - quantification of lysosome association with CD8+ T cells internalized within BEC after 4 hand 24 h co-culture periods. N=2. Both repeats showed robust lack of association with lysosomes so N=2 was deemed sufficient to make this conclusion.

Figure 5D - quantification of internalised 48 h-activated CD8+ T cells per 100 BEC following 4 hr co-culture, following treatment with anti-CD103 antibody treatment. N=1. The data generated from this experiment was negative and is displayed to show the lack of effect of this antibody.

Figures 8B-E - comparisons of E-cadhern+ and E-cadherin- cells isolated using flow activated cell sorting. N=2. Experimental number was limited to two because of large differences in internalization (70% difference) which was reproduced with each experiment.

Figure 8F - treatment of E-cadherin+ T cells with small molecular inhibitors. N=1. This experiment was limited to one due to the limited amount of cells retrieved after sorting and because the experiment was previously performed with more repeats using unsorted cells.

For fluorescence microscopy images (immunocytochemistry and immunohistochemistry), representative images used for figures were selected from a range (minimum of three) captured for each individual sample which showed the same observation. For electron microscopy, images selected for figures were chosen from a pool of images acquired from images captured across two biologically independent experiments.

Each attempt at replication was successful.

Randomization

For co-culture experiments (both quantitative and descriptive) the allocation of wells used for experimental parameters was not randomized, but covariates were controlled by repeating experimental conditions in triplicate, in which a mean value was later used for analysis. The source of T cells used within co-culture experiments, which did not involve the use of PBC-derived samples) were selected based on availability of blood at the time of experiment. BEC were selected at random from frozen stocks or from cultures that were in the process of passage. BEC were not used when beyond passage four.

For the selection of biological cases used for immunohistochemistry and flow cytometry phenotyping, a random selection of pre-anonymised cases were selected from out local biological samples bank. Samples were allocated onto different groups based on the diagnosis (either PBC or HFE in case of flow cytometry experiment or PBC, donor livers or other chronic disease in case of the semi-quantitative tissue analysis).

Blinding

Blinding was not required for any experiments that were analysed using automated software or where only two experiment parameters were compared. Blinding was also not required when gathering data using flow cytometry as the effect of an experimental parameter was not being assessed. Additionally, samples were processed upon their receipt from the clinic, so it was not possible to blind samples for the disease that they were associated with. Semi-quantitative analysis of tissue staining was blinded by covering all information on microscopy slides which revealed their disease of origin. Blinding of experimental conditions for co-culture experiments was performed when quantifying internalization events manually.

For descriptive assessments of images captured for immunocytochemistry (ICC) or electron microscopy, blinding was not possible as the person conducting the experiments were also the same individuals performing image acquisition. Additionally, only single full stained and IMC-stained conditions were performed at any one time in the case of ICC. For EM, only a single co-culture specimen and matched BEC-alone condition were imaged in any one session.

Reporting for specific materials, systems and methods

We require information from authors about some types of materials, experimental systems and methods used in many studies. Here, indicate whether each material, system or method listed is relevant to your study. If you are not sure if a list item applies to your research, read the appropriate section before selecting a response.

Materials & experimental systems

n/a	Involved in the study	
☐	☒	Antibodies
☒	☐	Eukaryotic cell lines
☒	☐	Palaeontology and archaeology
☒	☐	Animals and other organisms
☒	☐	Clinical data
☒	☐	Dual use research of concern
☒	☐	Plants

Methods

n/a	Involved in the study	
☒	☐	ChIP-seq
☐	☒	Flow cytometry
☒	☐	MRI-based neuroimaging

Antibodies

Antibodies used

Here we provide all targets, species, manufacturer, clone, and catalogue numbers of all antibodies used throughout this report (round brackets show clone, square brackets show company and catalogue numbers, curled brackets show lot numbers). For some antibodies, multiple lots were used (denoted as "multiple").

The following antibodies were used for flow cytometry analysis:

BV510 α-hCD3 (OKT3) [Biolegend - 317332] {multiple lots}, BUV395 α-hCD4 (SK3) [BD Biosciences 564724] {multiple lots}, BV605 α-hCD8 (SK1) [BioLegend 337217] {multiple lots}, APC α-hE-cadherin (67A4) [BioLegend 324108] {multiple lots}, BV711 α-hCD45RA (HI100) [BioLegend 304130] {lot B345247}, PE-594 α-hCCR7 (150503) [BD 562381] {2199957}, PE-Cy7 α-hCD107a (H4A3) [BioLegend 328617] {B284259}, PE α-hCD69 (FN50) [Biolegend 310906] {B352653}, BV786 α-hCD103 (Ber-ACT8) [BD Biosciences 743654] {2250662}, FITC α-hKLRG1 (SA231A2) [BioLegend 367714] {B320897}, AF700 α-hCXCR3 (G025H7) [Biolegend 353742] {B330851}, PE α-hCCR6 (11A9) [BD 559562] {7019800}, APC α-hCD161 (HP-3G10) [BioLegend 339912] {B302178}, BV421 α-hCXCR6 (K041E5) [BioLegend 356014] {multiple lots}, BUV395 α-hCD27 (L128) [BD 563815] {7004623}, PerCP α-hCD57 (HNK-1) [Biolegend 359622] {B321641}, BV650 α-hCD28 (CD28.2) [Biolegend 302946] {B315410}, PE α-hβ-catenin (12F7) [BioLegend 862604] {B303685}, AF700 hLFA-1 (m24) [BioLegend 363422] {B283404}, APC α-hCD49a (TS2/7) [BioLegend 328314] {B270613}, PerCP Cy5.5 α-hPerforin (dG9) [BioLegend 308114] {B282783}, and PerCP Cy5.5 α-hGranzyme B (QA16A02) [BD 561142] {B357972}. All antibodies were purchase from Biolegend, eBioscience (Thermo Fisher) or BD Bioscience.

APC-E-cadherin, BV786-CD103 and BV605-CD8 were also used to sort live E-cadherin-hi/lo CD8+ T cells, using APC-mIgG1 as an isotype-matched control to assist gating.

The following unconjugated primary antibodies were used for immunohistochemistry:

α-hCD3 mouse IgG1 (F7.2.38) [Abcam ab17143] {GR3395244-2}, α-hCD4 mouse IgG2a (OTI5D9) [Novus Bio NBP2-46149] {Multiple lots}, α-hCD8 mouse IgG2b (4B11) [Invitrogen/Thermo Fisher MA1-80231] {Multiple lots}, α-hCD69 mouse IgG1 (8B6) [Invitrogen/Thermo Fisher MA5-15612] {WI3371233}, α-hCD103 Rabbit IgG (EPR22590-27) [Abcam ab224202] {multiple lots], α-hKLRG-1 rabbit IgG (2388C) [R&D systems MAB7029] {CLPU0221081}, α-hE-cadherin mouse IgG2a (36/E-cadherin) [BD Biosciences 610182] {Multiple lots}, α-hβ-catenin mouse IgG1 (12F7) [Biolegend 844608] {B305601}, α-hCytokeratin-19 rabbit IgG (EP1580Y) [abcam ab52625] {GR3255534-19}, α-hCytokeratin-19 mouse IgG1 (1H6) [Invitrogen/Thermo Fisher MA515862] {YH4011082A}, α-hEpCAM mouse IgG1 (EGP40/1372) [abcam ab218448] {GR3417029-2}.

The following primary antibodies were used for immunocytochemistry:

α-hE-cadherin mouse IgG2a (36/E-cadherin) [BD Biosciences 610182] {Multiple lots}, α-hβ-catenin mouse IgG1 (12F7) [Biolegend 844608] {B305601}, α-hCytokeratin-19 rabbit IgG (EP1580Y) [abcam ab52625] {GR3255534-19}, α-hα-tubulin mouse IgG1 (TU-01) [Invitrogen/Thermo Fisher MA1-19162] {543170} and α-hEpCAM (HEA-125) [Progen Biotechnik 61004) {703131B}.

The following unconjugated isotype-matched controls were used for both immunohistochemistry and immunocytochemistry:

ULTRA-LEAF purified mouse IgG1 isotype control (MOPC-21) [Biolegend 400166] {B317345}, ULTRA-LEAF purified mouse IgG2a isotype control (MG2a-53) [Biolegend 41508] {B304626}, ULTRA-LEAF purified mouse IgG2b isotype control (MG2b-57) [Biolegend 401216] {B297109}, Rabbit mAb XP(R) isotype control (DA1E) [Cell Signalling Tech 3900S] {50}.

The following primary antibodies were used for western blotting:

α-Phospho-hCofilin Ser3 rabbit IgG [Invitrogen/ThermoFisher 44-1072G] {2456324}, α-hCofilin mouse IgG2a (GT567) [Invitrogen/ThermoFisher MA5-17275] {YG3988356), α-hβ-actin mouse IgG1 (Merck; AC-15, A5441) {0000126949}.

Fluorophore conjugated secondary antibodies used for western blotting, ICC and IHC are as follows:

Dylight 488 horse α-rIgG [DI-1088] {ZH0423}, AF488 goat α-rIgG [A-11008] {1981125}, AF488 goat α-mIgG1 [A21121] {2339820}, AF488 goat α-mIgG2a (A21131) {2273777}, goat AF546 goat α-mIgG2a [A21133] {2447873}, Dylight 594 horse α-rIgG [DI-1094] {ZH0527}, AF647 goat α-mIgG1 [A21240] {2652974}, AF647 goat α-mIgG2b [A21242] {2273692}, and AF647 plus α-rIgG [A32733] {WL333739}. All Dylight-conjugated antibodies were purchased from Vector Laboratories through 2BScientific. All AF-conjugated antibodies were purchased from ThermoFisher Scientific (Invitrogen/Thermo Fisher).

Validation

All antibodies were selected based on manufacturers verification of human reactivity for their required purpose.

The majority of antibody clones used for flow cytometry panels were selected based on those used previously by our authors here: Oo et al, J Immunol, 2010. Pe-Cy7 CD38 from eBioscience was validated for the staining of CD8+ T cells previously: Fernandez et al, Stem Cells, 2014. Others were chosen based on manufacturer statement of quality control and the provision of figures showing reactivity against human peripheral blood mononuclear cells on manufacturers websites, with the exception of APC-E-cadherin which was validated in our experiments using fluorescence minus-one (FMO) staining panels, replacing this antibody with an isotype-matched control (Main text figure 6B). The majority of flow cytometry antibodies were purchased from either Biolegend or BD Biosciences which have the following quality control statements:

Biolegend: "Specificity testing of 1-3 target cell types with either single- or multi-color analysis (including positive and negative cell types). Once specificity is confirmed, each new lot must perform with similar intensity to the in-date reference lot. Brightness (MFI) is evaluated from both positive and negative populations. Each lot product is validated by QC testing with a series of titration dilutions.". Link: https://www.biolegend.com/en-gb/quality/quality-control

BD Biosciences: "The specificity is confirmed by using multiple applications that may include a combination of flow cytometry, immunofluorescence, immunohistochemistry or western blot to test a combination of primary cells, cell lines or transfectant models.

All flow cytometry reagents are titrated on the relevant positive or negative cells. To save time and cell samples for researchers, pre-titrated test size reagents are bottled at an optimal concentration, with the best signal-to-noise ratio on relevant models." Link: https://www.bdbiosciences.com/en-gb/products/reagents/flow-cytometry-reagents/research-reagents/quality-and-reproducibility.

All flow cytometry antibodies were also titrated using human peripheral blood mononuclear cells prior to their experimental use.

Antibodies used for immunohistochemistry that had been validated previously by our authors are as follows:

α-hCD4 mouse lgG2a (Novus Bio; OTISD9; NBP2-46149) - Wiggins et al, Gut, 2022
α-hE-cadherin mouse lgG2a (BD Bioscience; 36/E-cadherin) - Davies et al, Cell Reports, 2019
α-hβ-catenin mouse lgGl (Biolegend; 12F7; 844608) - Davies et al, Cell Reports, 2019

Antibodies used for immunocytochemistry that had been validated previously by our authors are as follows:

α-hE-cadherin mouse lgG2a (BD Bioscience; 36/E-cadherin) - Davies et al, Cell Reports, 2019
α-hβ-catenin mouse lgG1 (Biolegend; 12F7; 844608) - Davies et al, Cell Reports, 2019

All new antibodies were selected based on human reactivity verification and both immunofluorescence and immunohistochemistry validation according to manufacturers websites, based on the provision of microscopy images and references where the antibodies had been used to stain human tissues and/or cells.

Antibodies were then titrated by performing single stains. For immunohistochemistry, formalin-fixed paraffin- embedded (FFPE) human liver tissue using chromagen-based detection; after primary antibody incubation, tissues were washed and incubated with ImmPRESS®HRP Universal Antibody (Horse Anti-Mouse/Rabbit lgG) Polymer Detection Kit (Vectorlabs; MP-7500). Tissue sections were then washed and stains were developed using lmmPACT®DAB Substrate Kit (Vectorlabs; SK-4105). All single stains were then inspected by a pathologist for accuracy (Dr Gary M Reynolds - listed author). For immunocytochemistry, antibodies were titrated by staining cells that would ultimately be the experimental target. Manufacturer validation statements for immunohistochemistry and immunocytochemistry are as follows:

Abcam: "IHC and ICC determine whether an antibody recognizes the correct protein based on cellular and subcellular localization. Antibody specificity is confirmed by looking at cells that either do or do not express the target protein within the same tissue. Initially, our scientists will review the available literature to determine the best cell lines and tissues to use for validation. We then check the protein expression by IHC/ICC to see if it has the expected cellular localization. If the localization of the signal is as expected, this

antibody will pass and is considered suitable for use in IHC/ICC. We use a variety of methods, including staining multi-normal human tissue microarrays (TMAs), multi-tumor human TMAs, and rat or mouse TMAs during antibody development. These high-throughput arrays allow us to check many tissues at the same time, providing uniformly as all tissues are exposed to the exact same conditions. We are currently working towards using KO cell lines for our ICC validation." Link: https://www.abcam.com/primary-antibodies/how-we-validate-our-antibodies#IHC%20and%20ICC.

Thermo Fisher: This company supplies all Invitrogen and Fisher Scientific products. They have seperate validation statements and protocols for immunohistochemistry (https://www.thermofisher.com/uk/en/home/life-science/antibodies/antibodies-learning-center/antibodies-resource-library/antibody-application-testing-protocols/immunohistochemistry-paraffin-protocol-application-testing.html) and immunocytochemistry (https://www.thermofisher.com/uk/en/home/life-science/antibodies/antibodies-learning-center/antibodies-resource-library/antibody-application-testing-protocols/immunofluorescence-protocol-adherent-suspension-application-testing.html).

For western blotting, α-hβ-actin mouse IgG1 (Merck; AC-15; A5441) was previously validated by our authors here: Wilkinson et al, iScience, 2023. Other antibodies used for western blotting were selected based on the provision of figures and references on the manufacturers website demonstrating their functionality for this purpose.

Plants

Seed stocks

N/A

Novel plant genotypes

N/A

Authentication

N/A

Flow Cytometry

Plots

Confirm that:

☒ The axis labels state the marker and fluorochrome used (e.g. CD4-FITC).

☒ The axis scales are clearly visible. Include numbers along axes only for bottom left plot of group (a 'group' is an analysis of identical markers).

☒ All plots are contour plots with outliers or pseudocolor plots.

☒ A numerical value for number of cells or percentage (with statistics) is provided.

Methodology

Sample preparation

Peripheral blood mononuclear cells (PBMCs) were isolated using a gradient strategy as previously described (Jeffery, H. C. et al, Journal of immunology, 2019). Briefly, blood was diluted 1:1 with phosphate-buffered saline (PBS) and gently layered on Lympholyte (Cedarlane) (2:1 ratio). After centrifugation, the mononuclear cells buffy coat was carefully removed and washed in RPMI three times. Cells were then resuspended in FACS buffer (2% fetal bovine serum, 2 mM EDTA in PBS) in round-bottom 96-well plates at concentration of 2xl07 cells/ml. Cells were incubated with Fe block for 10 min at 4°C. Cells were washed and resuspended in 100 ul of appropriate antibody cocktail diluted in cold FACS Buffer. Cells were incubated for 25 minutes at 4°C. Cells were washed twice in ice-cold FACS Buffer and resuspended in 100 µL Cytofix (BD Biosciences), then incubated at room temperature in the dark for at least 20 min. Cells were washed twice in cold FACS Buffer then resuspended in lO0uL of appropriate intracellular antibody cocktail made up lX Perm buffer (lnvitrogen). Cells were incubated overnight (at least 16 h) at room temperature in the dark. Cells were washed once in Perm buffer then twice in cold FACS buffer before being resuspended in cold FACS Buffer and transferred to 5 ml round-bottom polystyrene tubes for analysis.

Instrument

All samples were acquired using an LSR Fortessa X20 (BD Biosciences) equipped with a UV laser.

Software

Flow cytometry data was acquired using FACSDiva (BD Biosciences) and analysed using FlowJo v10.5.1.

Cell population abundance

Abundance and purity of post-sort fractions was determined by relevant staining using flow cytometry analysis. Purity was greater than 95% on an acquisition of at least 10000 events.

Gating strategy

Gating strategies are indicated in the figures in the manuscript. Briefly all the FSC/SSC gate were set to remove cell debris and dead cells (small FSC v SSC) and large clumps or aggregates of cells (large FSC or SSC) and used consistently across all samples. A lymphocyte gate consistent across all the samples was drawn to exclude monocytes from the downstream gates. Singlets were gated using FSC-H vs FSC-A. CD3-BV510 vs 7AAD viability dye were used to gate on CD3+7AAD- as viable T cells. CD4- BUV395 vs CD8 BV- 605 was used to gate on TCD4+ and TCD8+ cells. These steps are shown in figure 3B. In figure 3C, a back gate was applied to show FSC and SSC of the activated and non-activated CD8+ T cells. In Figure 5 and supplementary

figure 6C+D, CD69-PE vs CD103 -BV785 was applied on CD8+ T cells and CD4+ T cells, respectively. In figure 6B, E-cadherin-APC+ CD8+ T cells are shown with both fluorescent histogram and contour plot compared to an FMO control. In Figure 6E CD69 - PE vs E-cadherin - APC gate was applied on CD8+ T cells. In figure 8, CD8-BV605 vs E-cadherin -APC gating strategy was used to sort E-cadherin-hi and E-cadherin-lo cells. In Supplementary figure 6A-B cells were gated as: CD8 - BV605 vs CCR6 - PE, CXCR3-AF700, CXCR6 - BV421, CD49a - APC. In supplementary figure 10C, post-sorted fraction (CD8+ E-cadherin+ T cells) were gated as CD103-BV785 vs SSC-A. All the gating for relevant markers were drawn using isotype-matched controls.

☒ Tick this box to confirm that a figure exemplifying the gating strategy is provided in the Supplementary Information.
