# Supplementary material for: Expression of E-cadherin by CD8+ T cells promotes their invasion into biliary epithelial cells
Source: Nat Commun. 2024 Jan 29;15:853. doi: 10.1038/s41467-024-44910-2 (PMC10825166; doi:10.1038/s41467-024-44910-2)
Supplement: Supplementary file 4 — Description of additional supplementary files [file 41467_2024_44910_MOESM4_ESM.docx]

**DESCRIPTION OF ADDITIONAL SUPPLEMENTARY FILES DOCUMENT**

**SUPPLEMENTARY SOFTWARE FILES**

**Supplementary software file 1**. CellProfiler analysis pipeline file used to quantify the association of internalized CD8+ T cells with lysosomes when using Lysotracker™ labelling.

**Supplementary software file 2**. CellProfiler analysis pipeline file developed in this investigation to quantify cellin-cell structures by labelled cells with CellTracker™ dyes and wheat germ agglutinin membrane labels.

**SUPPLEMENTARY MOVIES**

**Supplementary Movie 1.** Time-lapse video of cocultured CD8+ T cells and biliary epithelial cells (BEC). Peripheral blood derived CD8+ T cells were activated with α-CD3/CD28 stimulation 48 h prior to co-culture with BEC. Labelled BEC (CellTracker™ Green; yellow) and peripheral blood derived CD8+ T cells (CellTracker™ Red; cyan) were imaged 1 h after initial co-culture. Cells were imaged every 4 min for 3 h using a Zeiss Cell Discoverer 7. Phase Gradient (grey) light microscopy images were acquired simultaneously. White arrow indicates T cell that is about to be internalised. Yellow arrow shows position of the same T cell at the end of the imaging period. See Supplementary Fig. 1.

**Supplementary Movie 2.** Multichannel 3Dreconstructed confocal micrographs of co-cultured CD8+ T cell and BEC showing complete T cell internalisation. Cells were fixed following 4 h co-culture and stained for cytokeratin-19 (CK19; yellow) and EpCAM (grey). Images show 3Dreconstructions of Z-stack confocal micrographs demonstrating labelled CD8+ T cell (Magenta) found enclosed within the CK19+ intermediate filament skeleton within a BEC, without any association with EpCAM (grey). Cells were permeabilised with 0.1% saponin prior to, and during, incubation with staining antibodies. Video shows successive 360-degree rotations of images displaying different channel combinations and matching 3D-volume renders. See Fig. 2D+E.

**Supplementary Movie 3**. Airyscan super-resolution orthographical confocal micrograph and multichannel 3D-reconstructed confocal micrographs of co-cultured CD8+ T cells and BEC labelled with phalloidin. Labelling was performed on co-cultured biliary epithelial cells (BEC; CellTracker™ green, yellow) and CD8+ T cells (bright Hoechst labelling, cyan) which were fixed after 4 h co-culture. Actin cytoskeleton was labelled using Alexa Fluor 594-conjugated phalloidin (magenta). Cells were permeabilised with 0.1% saponin prior to labelling. Video shows successional confocal planes of a Z-stack acquisition followed by successive 360-degree rotations of images displaying different channel combinations and matching 3D-volume renders Images demonstrate internalised CD8+ T cells and their respective actin cytoskeletons located above the basal skeleton of the BEC. See Fig. 2F.

**Supplementary Movie 4**. Immunohistochemistry staining of 50 μm-thick primary biliary cholangitis liver tissue section showing CD103+ CD8+ T cell internalised within BEC. Video demonstrates transition between original image and 3D-volume rendering and zooms into region CD103+ (magenta) CD8+ (green) T cell is fully internalised within E-cadherin+ (grey) cytokeratin-19+ (CK19; orange) biliary epithelial cells (BEC). See Supplementary Fig. 8A.

**Supplementary Movie 5.** Immunohistochemistry staining of primary biliary cholangitis liver tissue section showing E-cadherin+ CD103+ CD8+ T cell. Video demonstrates transition between original image and 3D-volume rendering, as well as the generation of new channel showing colocalization between Ecadherin (orange) and βcatenin (green). Video zooms into BEC-CD8+ (white) T cell interface and shows overlap between the colocalisation channel (grey) and CD103 (magenta). See Fig. 7C.

**Supplementary Movie 6**. Immunocytochemistry staining showing E-cadherinβ-catenin interactions formed between CD8+ T cells and biliary epithelial cells (BEC). Immunocytochemistry (ICC) staining for E-cadherin (yellow) and β-catenin (magenta) of BEC co-cultured with 48 h activated CD8+ T cells (CellTracker™ Red, orange). Cells were cocultured for 4 h prior to fixation and staining. Video demonstrates transition between original image and 3D-volume rendering, as well as the generation of a new channel showing colocalisation between Ecadherin and β-catenin. See Fig. 7D.

**Supplementary Movie 7**. Airyscan super-resolution orthographical confocal micrographs of PBC patient blood-derived CD8+ T cells and BEC stained by immunocytochemistry (ICC). Cells were fixed following 4 h co-culture and stained for cytokeratin-19 (CK19; grey) and α-tubulin (yellow). Video shows images of successional confocal planes of a Z-stack acquisition. Overall image shows attached and internalised CD8+ T cells, including one undergoing cell division. Cells were permeabilised with 0.1% saponin prior to, and during, incubation with staining antibodies. See Fig. 9D.
